# Construction of a screening system for lipid-derived radical inhibitors and validation of hit compounds to target retinal and cerebrovascular diseases

**DOI:** 10.1016/j.redox.2024.103186

**Published:** 2024-05-08

**Authors:** Ryota Mori, Masami Abe, Yuma Saimoto, Saki Shinto, Sara Jodai, Manami Tomomatsu, Kaho Tazoe, Minato Ishida, Masataka Enoki, Nao Kato, Tomohiro Yamashita, Yuki Itabashi, Ikuo Nakanishi, Kei Ohkubo, Sachiko Kaidzu, Masaki Tanito, Yuta Matsuoka, Kazushi Morimoto, Ken-ichi Yamada

**Affiliations:** a Department of Molecular Pathobiology, Faculty of Pharmaceutical Sciences, Kyushu University, 3-1-1 Maidashi Higashi-ku, Fukuoka, 812-8582, Japan; b Department of Drug Discovery Structural Biology, Faculty of Pharmaceutical Sciences, Kyushu University, 3-1-1 Maidashi Higashi-ku, Fukuoka, 812-8582, Japan; c Institute for Open and Transdisciplinary Research Initiatives, Osaka University, 1-6 Yamadaoka, Suita, Osaka, 565-0871, Japan; d Quantum RedOx Chemistry Team, Institute for Quantum Life Science (iQLS), Quantum Life and Medical Science Directorate (QLMS), National Institutes for Quantum Science and Technology (QST), 4-9-1 Anagawa, Inage-ku, Chiba, 263-8555, Japan; e Institute for Advanced Co-Creation Studies, Osaka University, 1-6 Yamada-oka, Suita, Osaka, 565-0871, Japan; f Department of Ophthalmology, Shimane University Faculty of Medicine, 89-1 Enya Izumo, Shimane, 693-8501, Japan

**Keywords:** Oxidized lipids, Radical-trapping antioxidants, Methyldopa, Retinal damage, Bilateral common carotid artery stenosis

## Abstract

Recent studies have highlighted the indispensable role of oxidized lipids in inflammatory responses, cell death, and disease pathogenesis. Consequently, inhibitors targeting oxidized lipids, particularly lipid-derived radicals critical in lipid peroxidation, which are known as radical-trapping antioxidants (RTAs), have been actively pursued. We focused our investigation on nitroxide compounds that have rapid second-order reaction rate constants for reaction with lipid-derived radicals. A novel screening system was developed by employing competitive reactions between library compounds and a newly developed profluorescence nitroxide probe with lipid-derived radicals to identify RTA compounds. A PubMed search of the top hit compounds revealed their wide application as repositioned drugs. Notably, the inhibitory efficacy of methyldopa, selected from these compounds, against retinal damage and bilateral common carotid artery stenosis was confirmed in animal models. These findings underscore the efficacy of our screening system and suggest that it is an effective approach for the discovery of RTA compounds.

## Highlights

• We developed a novel high-throughput screening technique targeting lipid radicals.
• This system is effective for the discovery of radical-trapping antioxidants.
• Efficacy of hit compounds for oxidative stress-related diseases has been reported.
• Methyldopa ameliorated pathological features of two disease models.

## Abbreviations

AAPH: 2,2′-azobis(2-methylpropionamidine) dihydrochloride
d-AMD: dry age-related macular degeneration
ANOVA: analysis of variance
BCAS: bilateral common carotid artery stenosis
BRB: blood–retinal barrier
CBF: cerebral blood flow
CCA: common carotid artery
CEPs: carboxyethylpyrroles
DCP: dihexadecyl phosphate
DFT: density functional theory
DPPH: diphenyl-2-picrylhydrazyl
ERG: electroretinography
FDA: Food and Drug Administration
FRAP: ferric reducing antioxidant power
MD: methyldopa
MG/MΦ: microglia/macrophages
MNU: *N*-methyl-*N*-nitrosourea
NBD-Pen: 2,2,6-trimethyl-4-(4-nitrobenzo[1,2,5]oxadiazol-7-ylamino)-6-pentylpiperidine-1-oxyl
NBD-TEEPO: 2,2,6,6-tetraethyl-4-(4-nitrobenzo[1,2,5]oxadiazol-7-ylamino)piperidine-1-oxyl
NORT: novel object recognition test
NSR: neurosensory retina
ONH: optic nerve head
ONL: outer nuclear layer
ORAC: oxygen radical absorbance capacity
PB: phosphate buffer
PUFAs: polyunsaturated fatty acids
ROS: reactive oxygen species
RPE: retinal pigment epithelium
RTAs: radical-trapping antioxidants

## 1 Introduction

Lipids, particularly phospholipids containing polyunsaturated fatty acids (PUFAs), are readily oxidized by oxidants to produce oxidized phospholipids. Recently, several studies have highlighted the ability of oxidized lipids and their protein-reactive derivatives to cause inflammation [1,2] and angiogenesis [3]. Consequently, there has been a surge in research to explore the in vivo regulation of these functions and their relevance to disease. In 2012, the concept of “ferroptosis,” a form of cell death triggered by iron-dependent generation of oxidized lipids, was proposed [4]. Ferroptosis plays a role in the pathogenesis of retinal diseases, neurodegenerative diseases, and ischemia-reperfusion injury [5]. Thus, oxidized lipids or their formation mechanisms may be valuable therapeutic targets in preventing disease onset or mitigating disease progression.

Oxidized lipids are products of chain reactions during lipid peroxidation, in which lipid radicals are pivotal molecules. Extensive research has been conducted to identify inhibitors that specifically target lipid radicals or oxidized lipids with the aim of preventing or treating diverse diseases. Numerous techniques have been developed and widely employed to measure lipid radicals or oxidized lipids, including diphenyl-2-picrylhydrazyl (DPPH), ferric reducing antioxidant power (FRAP), and oxygen radical absorbance capacity (ORAC) [6]. Nevertheless, the inhibitors identified using these detection methods do not consistently yield the anticipated outcomes, and conflicting results have been reported [7]. However, numerous studies have documented the accumulation of oxidized lipids in animal experiments and clinical samples, indicating the role of lipid-derived radicals in the pathogenesis of specific diseases. We believe that this discrepancy stems from the lack of suitable screening methods for inhibitor identification.

Lipid radicals are lipid-soluble molecules. In addition, the in vivo environment (cell and organelle membranes), in which lipid radicals are generated, is also lipophilic. However, the above-mentioned screening method targets reactions with oxidants, including reactive oxygen species (ROS), necessitating the use of a water-soluble environment for the screening system. ROS and lipid radicals/oxidized lipids are water- and lipid-soluble compounds, respectively, and a screening method must be able to separate them properly. It is possible to propose suitable inhibitors only when a system that can be appropriately evaluated in a lipid-soluble environment is available.

Recently, a remarkable probe called styrene-conjugated BODIPY chromophore (STY-BODIPY), which is a modified version of C11-BODIPY^581/591^, a widely used probe in ferroptosis research, was reported [8]. This probe shows a significant fluorescence shift upon reaction with peroxyl radicals. In this study, the authors initiated lipid peroxidation in liposomes using azo compounds as radical chain initiators and evaluated the generation of lipid radicals by measuring the fluorescence shift of STY-BODIPY. They screened approximately 30 compounds, including phenolic compounds, and found a good correlation between the inhibition rate constants for peroxyl radicals of STY-BODIPY in liposomes and the IC_50_ values for RSL-3-induced ferroptosis in Pfa1 MEF cells.

In contrast, we previously focused on nitroxides that possess intramolecular unpaired electrons and remain stable in both water and lipid environments. We further modified the substituents around the N–O moiety of the nitroxide to achieve reaction specificity toward lipid radicals and avoid unfavorable reactions with antioxidants [9]. We developed the fluorescent probe 2,2,6-trimethyl-4-(4-nitrobenzo [1,2,5]oxadiazol-7-ylamino)-6-pentylpiperidine-1-oxyl (NBD-Pen) [10]. The fluorescent NBD-Pen is turned ON by reacting with lipid radicals; therefore, NBD-Pen acts as a radical-trapping antioxidant (RTA). This probe selectively detects lipid-derived radicals over other reactive species, such as ROS, in biological systems. Additionally, this probe offers valuable benefits for biological experiments, as it is suitable for cell imaging, staining of tissue sections [10], and structural analysis of lipid radicals combined with the probe through radical reactions [11].

The requirements of an ideal screening system for inhibitory compounds targeting lipid radicals warrant further study. The system must exhibit high reaction selectivity and sensitivity toward lipid radicals, show a significantly fast second-order rate constant, and mimic the environment in which lipid radicals are generated. In actual experiments, a competitive reaction between library compounds and lipid radical detection probes for lipid radicals is expected. Therefore, the detection probes must have large secondary reaction rate constants for lipid radicals. The reaction rate constant between C11-BODIPY and peroxyl radicals has been estimated to be 6.0 × 10^3^ M^−1^s^−1^ [12]. By comparison, the previously reported rate constant for the reaction of TEMPO, the most representative nitroxide, with carbon-centered radicals is approximately 10^7−9^ M^−1^s^−1^ [13]. Other screening requirements include reliability, reproducibility, and high-throughput capabilities.

We have previously reported that substitution at the α-position of piperidine nitroxides affects their reactivity with reducing antioxidants by altering the steric and electronic environments around the N–O moiety [14] and that the inhibitory activity of nitroxides against lipid peroxidation depends on their lipophilicity [15]. In this study, we synthesized the fluorescent probe 2,2,6,6-tetraethyl-4-(4-nitrobenzo [1,2,5]oxadiazol-7-ylamino)piperidine-1-oxyl (NBD-TEEPO), which exhibits minimal reactivity with antioxidants and displays high specificity for lipid radicals. Subsequently, we established a chemical screening system to evaluate the inhibitory activity against lipid radicals and assessed compounds from a United States Food and Drug Administration (FDA)-approved drug library using the NBD-TEEPO probe. Through this screening, we identified several compounds, and further searches of PubMed to identify hit compounds revealed their use as repurposed compounds, not only for various animal disease models but also for patients. These results demonstrated the validity of the screening system and the potential of lipid radicals as targets in related diseases. Furthermore, to validate our methodology, we selected methyldopa (MD) from the identified hit compounds for evaluation in several animal disease models, including those for retinal damage and vascular dementia.

## 2 Materials and methods

### 2.1 Animals

All procedures and animal care were approved by the Committee on Ethics of Animal Experiments, Graduate School of Pharmaceutical Sciences, Kyushu University (permit number: A21–151, A21–152, A23–121, A23-147) and conducted according to the Guidelines for Animal Experiments of the Graduate School of Pharmaceutical Sciences, Kyushu University.

Eight-week-old male BALB/c and ddY mice were purchased from SLC Inc., and 10–12-week-old (weighing 24–29 g) male C57BL/6J mice were purchased from Jackson Laboratory Japan (Kanagawa, Japan). All mice were maintained under a dim 12 h light-dark cycle (lights on from 7:00 to 19:00) with access to a standard diet (CLEA Japan, Tokyo, Japan) and water *ad libitum*.

### 2.2 Chemicals

NBD-Pen and NBD-TEMPO were prepared as previously reported [10]. The method used for NBD-TEEPO synthesis is included in the Supplementary Material.

### 2.3 Liposome preparation

Egg phosphatidylcholine (Egg PC) was weighed (200 mg) in a dry 200 mL eggplant flask and dissolved in 2 mL chloroform. Dihexadecyl phosphate (DCP) (8 mg) was dissolved in 2 mL chloroform and 1 mL methanol was added to the flask. The solvent was evaporated for 30 min and vacuum-dried for 10 min to form a lipid film at the bottom of the flask. The film was hydrated with 10 mL phosphate buffer (PB; 10 mM, pH 7.4), yielding 20 mg/mL Egg PC and 0.8 mg/mL of a DCP liposome suspension. The liposome suspension was then extruded 10 times using a mini extruder (Avanti Polar Lipids, Alabaster, AL, USA) equipped with a 100 nm polycarbonate membrane (Cytiva, Tokyo, Japan).

### 2.4 Evaluation of the reactivity of fluorescent nitroxide probes with reductants

In a 96-well black/clear bottom plate (Thermo Fisher Scientific, Waltham, MA, USA), fluorescent nitroxide probe (5 μM) and 50 μM of various reductants (ascorbic acid, uric acid, chlorogenic acid, edaravone, ferrostatin-1, Trolox, vitamin E) were mixed with liposomes (2.5 mg/mL Egg PC and 0.1 mg/mL DCP) in PB (10 mM, pH 7.4), sealed, and incubated at 37 °C for 45 min. Fluorescence intensity was measured using an EnSpire Multimode Plate Reader (PerkinElmer, Waltham, MA, USA) with excitation at 470 nm and fluorescence wavelength of 530 nm from bottom.

### 2.5 Assay of lipid peroxidation using NBD-TEEPO in liposomes

NBD-TEEPO (5 μM), liposomes (2.5 mg/mL Egg PC and 0.1 mg/mL DCP), and PB (10 mM, pH 7.4) were mixed in a 96-well black/clear bottom plate (Thermo Fisher Scientific). Lipid peroxidation was initiated by mixing 2,2′-azobis(2-methylpropionamidine) dihydrochloride (AAPH) (20 mM) or FeSO_4_ (500 μM). The plates were then sealed and incubated at 37 °C for 45 min. The fluorescence intensity was measured using an EnSpire Multimode Plate Reader with excitation at 470 nm and fluorescence wavelength of 530 nm from the bottom.

### 2.6 Theoretical calculations

Density functional theory (DFT) calculations were performed using Gaussian 16 (Revision C.02; Gaussian Inc., Wallingford, CT, USA) [16]. The calculations were performed using a 52-processor HPC5000-XIL216TS-D8 at the B3LYP/6-311+G(d,p) level of theory [[17], [18], [19]]. Graphical outputs of the computational results were generated using the *GaussView* software program (ver. 6.1.1) developed by Semichem Inc [20].

### 2.7 LC/MS/MS analysis of NBD-TEEPO and lipid radical adducts

AAPH (10 mM) or FeSO_4_ (0.1 mM) + ascorbic acid (0.5 mM) was added to the mixtures of arachidonic acid (500 μM) and NBD-TEEPO (5 μM) in PB. After incubation for 2 h at 37 °C, the lipids were extracted using the modified Bligh and Dyer method [21]. Briefly, 1 mL methanol and 1 mL chloroform containing 100 μM dibutyl hydroxytoluene and 100 μM ethylenediaminetetraacetic acid were added to 1 mL sample PB solution. The extracted solution was dried under a stream of nitrogen gas, and the residue was dissolved in methanol (200 μL).

For the analysis of NBD-TEEPO and lipid radical adducts, LC/MS/MS was performed using an LCMS-8060 (Shimadzu). The mass spectrometer was equipped with an electron spray ionization source. The LC conditions were as follows: injection volume, 5 μL; autosampler temperature, 4 °C; column, InertSustain C18 column (2.1 × 150 mm, particle size of 3 μm, GL Sciences); column temperature, 40 °C; mobile phase, 5 mM ammonium formate in acetonitrile/H_2_O (2:1, v/v) (A) and 5 mM ammonium acetate in isopropanol/H_2_O (95:5, v/v) (B); flow rate, 0.4 mL/min; and gradient, 0%–100 % B, 0–20 min; 100 % B, 20–25 min; and 0 % B, 25–30 min. MS analysis was performed in the negative-ion mode, and MRM transitions for individual radical adducts are described in Table S1. The LC/MS/MS analysis was performed using LabSolutions (Shimadzu).

### 2.8 Compound screening using NBD-TEEPO in liposomes

An FDA-approved drug library (766 compounds) was used to screen for lipid radical inhibitors. NBD-TEEPO (5 μM) and liposomes (2.5 mg/mL Egg PC and 0.1 mg/mL DCP) in PB (10 mM, pH 7.4) were mixed in a 384-well clear bottom plate (Greiner BIO-ONE, Frickenhausen, Germany). Library compounds (10 μM) were added, and lipid peroxidation was initiated by mixing with AAPH (20 mM) or FeSO_4_ (500 μM). The plates were sealed and incubated at 37 °C for 45 min. The fluorescence intensity was measured using an EnSpire Multimode Plate Reader with excitation at 470 nm and fluorescence wavelength of 530 nm from the bottom. The activity values were determined as described below:

Activity values = 1 − (FL_initiator+compound_ −FL_background_) / (FL_initiator_ − FL_background_)

### 2.9 Light-induced retinal degeneration and drug administration

The BALB/c mice were exposed to damaging light as described previously [22]. Briefly, pupils were dilated with 0.5 % tropicamide and 0.5 % phenylephrine hydrochloride eye drops before exposure to light. Unanesthetized mice were exposed to 8000 lux of white fluorescent light for 10 h in wire-topped cages, and each cage contained one mouse. All mice were returned to a dim cyclic light environment after light exposure. For initial evaluation of the retinoprotective effects of the screening hit compounds, 50 μmol/kg of each compound (edaravone, fenoldopam, indapamide, dobutamine, or methyldopa [MD]) was administered intraperitoneally 30 min before light exposure. MD (Tokyo Chemical Industry, Tokyo, Japan) dissolved in saline was administered orally at 100 or 200 μmol/kg 30 min before light exposure, while the control and light-vehicle groups were administered saline orally. For eye drop administration, one drop of 0.45 or 1 % (w/v) MD dissolved in saline was applied to both eyes 30 min before and 5 h after light exposure. BCH (Sigma-Aldrich, St Louis, MO, USA) was dissolved in saline and administered orally at 200 mg/kg. Yohimbine (Tokyo Chemical Industry) was dissolved in purified water to 10 mg/kg, diluted to 5 mg/kg with 2 × PBS immediately before administration, and administered intraperitoneally 20 min before MD administration.

### 2.10 N-methyl-N-nitrosourea (MNU)-induced retinal degeneration and drug administration

MNU-induced retinal degeneration in ddY was induced via intraperitoneal administration of 40 mg/kg MNU dissolved in saline containing 0.05 % acetic acid, as previously reported [23]. Five days after administration, the eyes were enucleated to measure the outer nuclear layer (ONL) thickness. MD dissolved in saline was administered orally at 400 μmol/kg 30 min before and 5 h after MNU administration, while the control and MNU-vehicle groups were administered saline orally.

### 2.11 Measurement of the ONL thickness

The eyes were enucleated seven days after light exposure. Cryosections (8 μm) were stained with H&E. For each section, digitized images of the entire retina were captured BZ-X810 (Keyence, Osaka, Japan) at × 20 magnification. ONL thickness was measured every 180 μm (light-induced retinal degeneration model) or 200 μm (MNU-induced retinal degeneration model) from the optic nerve head (ONH) using BZ-X810 Analyzer (Keyence).

### 2.12 Electroretinography (ERG)

Flash ERGs were recorded seven days after light exposure using an ERG recording system (LS-W; Mayo Corporation, Aichi, Japan). The mice were dark-adapted overnight before measurement. Anesthesia was induced by intramuscular injection of a mixture of ketamine (100 mg/kg) and xylazine (20 mg/kg). The pupils were dilated using 0.5 % tropicamide and 0.5 % phenylephrine hydrochloride eye drops. LED electrodes were placed in both eyes. An identical reference electrode was placed on the mouth, a ground electrode was placed on the tail. A single flashlight (50 cd･s/m^2^) from an LED was used as the light stimulus. The a-wave amplitude was measured as the difference in voltage between the baseline value before the flash and the peak of the a-wave. The b-wave was measured as the difference in voltage between the peaks of the a-wave and b-wave. The a-wave and b-wave amplitudes obtained from the right and left eyes were averaged in each mouse.

### 2.13 Fluorescence imaging of retinal tissue sections

After light exposure, NBD-Pen (5.0 μmol/kg body weight in PBS containing 50 % PEG300) was administered intraperitoneally [22]. After 30 min, the eyes were enucleated. Cryosections (16 μm) were dried at room temperature for 15 min, mounted with ProLong Diamond antifade reagent with DAPI (Invitrogen, Carlsbad, CA, USA), and imaged using confocal microscopy on a Zeiss LSM700 (Carl Zeiss, Jena, Germany). ImageJ software was used to measure fluorescence intensity.

### 2.14 Immunohistochemistry

Cryosections (12 μm) were incubated at room temperature in a blocking solution (5 % normal donkey serum, 0.3 % Triton X-100 in PBS) for 1 h. Subsequently, retinal sections were incubated with primary antibodies at 4 °C overnight. The following primary antibodies were used: rabbit anti-C3 (Abcam, Cambridge, UK), rabbit anti-IBA1 (Wako, Osaka, Japan), rat anti-CD68 (Bio-Rad, Hercules, CA, USA), goat anti-GFAP (Abcam), and mouse anti-FDP-lysine (ACR-modified protein; a gift from Dr. Uchida, University of Tokyo, Japan), mouse anti-GS (glutamine synthase; Abcam) antibody. After washing, sections were incubated with secondary antibodies conjugated to Alexa Fluor 488 or Alexa Fluor 594 (Invitrogen) for 1 h at room temperature. Sections were mounted using Vibrance Antifade Mounting Medium with DAPI (Vector Laboratories, Burlingame, CA, USA) and analyzed using BZ-X810.

### 2.15 Microglia isolation

The retinas were digested with 1.5 mg/mL collagenase A (Roche, Basel, Switzerland) and 0.4 mg/mL DNase I (Sigma-Aldrich) for 30 min at 37 °C in HBSS. Single-cell suspensions were generated by passing the solution through 70 μm filters. Sorting was performed using an autoMACS Pro system (Miltenyi Biotec, Bergisch Gladbach, Germany) according to the manufacturer's instructions. Briefly, to purify microglia/macrophages (MG/MΦ), single-cell suspension was incubated with anti-CD11b-microbeads.

### 2.16 Retinal pigment epithelium (RPE) cell isolation

RPE cells were isolated according to the method reported by Shang et al. [24], with some modifications. The enucleated eyes were digested for 35 min at 37 °C in dispase II solution (2 % dispase II [Thermo Fisher Scientific], 10 mM HEPES [pH 7.4], 30 mM NaCl in DMEM [4.5 g/L glucose]). Digested eyeballs were washed twice with growth medium (DMEM [4.5 g/L glucose] containing 10 % FBS, 100 units/mL penicillin, 100 μg/mL streptomycin, and 2.5 mM l-glutamine). An incision was made near the ora serrata of the eyes and the anterior segment of the eye was removed. The remaining posterior eye was transferred to a new growth medium, and the neurosensory retina (NSR) was removed. The RPE–choroid complex was transferred to a new growth medium and RPE cell sheets were peeled from the choroid. Isolated RPE cells were washed twice with growth medium and centrifuged (800×*g*, 4 °C, 5 min).

### 2.17 TUNEL assay

Cryosections (8 μm) were labeled using an *in situ* cell death detection kit, TMR Red (Roche Diagnostics), according to the manufacturer's protocol. The sections were mounted with ProLong Gold antifade reagent with DAPI (Invitrogen) and analyzed using BZ-X810.

### 2.18 RNA extraction and quantitative RT-PCR

Total RNA was extracted from the frozen samples using ISOSPIN Cell & Tissue RNA (Nippon Gene, Tokyo, Japan), and reverse-transcribed using ReverTra Ace qPCR RT Master Mix (TOYOBO, Osaka, Japan); however, QuantAccuracy, RT-RamDA cDNA Synthesis Kit (TOYOBO) was used for isolated MG/MΦ. The expression level of mRNA was measured using THUNDERBIRD SYBR qPCR Mix (TOYOBO), and CFX Connect Real-Time PCR Detection System (Bio-Rad) was used with the following thermal cycle conditions: one cycle of 95 °C for 60 s, followed by 40 cycles of 95 °C for 15 s and 60 °C for 60 s, with a final stage of melting curve analysis. *Gapdh* or *Idh3b* were used for normalization, and relative gene expression was calculated using the 2^−ΔΔCt^ method. The primers used in this study are listed in Table S2.

### 2.19 Bilateral common carotid artery stenosis (BCAS) and drug administration

C57BL/6J mice were subjected to the BCAS procedure by attaching microcoils (specifications: piano wire diameter 0.08 mm, coiling pitch 0.5 mm, total length 2.5 mm, inner diameters 0.16 mm and 0.18 mm, purchased from Samini, Shizuoka, Japan), with some modifications from a previous report [25]. The mice were anesthetized with 2–3% isoflurane and maintained on 1.5 % isoflurane using a face mask. After a midline skin incision, the common carotid artery (CCA) was exposed and isolated bilaterally. Then, microcoils (0.16 mm for the right CCA and 0.18 mm for the left CCA) were applied to each CCA. After application, the incised region was sutured. The treated mice were observed until awakened and then allowed to feed and water *ad libitum*. In sham mice, the same procedure was performed, except for CCA stenosis.

The BCAS mice were divided into three groups: (1) vehicle, (2) MD 88.7 μmol/kg, and (3) MD 177.3 μmol/kg. The solvent used was 50 % PEG300 in PBS. MD was administered orally 30 min before BCAS and five times weekly after BCAS.

### 2.20 Measurement of cerebral blood flow (CBF)

CBF was measured using laser Doppler flowmetry (Laser Doppler ALF21; ADVANCE, Tokyo, Japan) without craniotomy and continuously monitored for 3–5 min before, immediately after, and 1, 3, 7, 14, and 28 days after BCAS. The measured area was 1 mm posterior and 2 mm lateral to the bregma. CBF values were presented as a percentage of the preoperative values.

### 2.21 Behavior analysis

A novel object recognition test (NORT) was performed to evaluate recognition memory. On the day before the test, the mice were habituated for 10 min in an environment with all conditions except the object. On the test day, mice were allowed to explore a cage containing two identical familiar objects (brown vials) for 5 min. After a 90-min interval, one of the objects was replaced by a novel object (colored block), which was explored for another 5 min. The discrimination index is the search time for a new object relative to the search times for both objects.

The Y-maze test was used to assess the spatial working memory and spontaneous activity. This test was performed 28 d after BCAS. Three arms were 41.5 cm long, 10 cm high, and 4 cm wide (YM-03 M; Muromachi Kikai, Tokyo, Japan). Each mouse was placed at the center, facing the same direction, and moved freely in the maze for 8 min. Successful alternations were evaluated as a percentage of the total number of arms entered during the 8 min period; mice that entered 14 or fewer arms during the 8 min period were excluded from the results.

### 2.22 Tissue collection and processing

All the mice were euthanized by cervical dislocation. The eyes were then enucleated and marked on the superior surface for orientation. Unfixed eyes were embedded in O.C.T. Compound (Sakura Finetek, Tokyo, Japan), and stored at −80 °C. For immunohistochemistry, the eyes were immediately fixed with 4 % PFA in PBS for 30 min. After removal of the cornea, the eyes were transferred to a 30 % sucrose in PBS solution and kept overnight at 4 °C. Next, the eyes were embedded and stored at −80 °C. Sagittal sections containing the whole retina, including the ONH, were prepared using a cryostat (Leica Microsystems, Wetzlar, Germany). To collect the retina, the cornea and lens were removed, and the retina was separated from the eyecup. The retina samples were immediately used for microglia isolation or frozen in liquid nitrogen and stored at −80 °C. The brains of the mice were removed immediately after euthanasia, and the olfactory bulb and cerebellum were excluded. The brains were postfixed in 4 % paraformaldehyde at 4 °C, embedded in paraffin, and sliced for Klüver-Barrera staining.

### 2.23 Protein extraction and western blotting

Hippocampal and corpus callosum tissues were homogenized in lysis buffer (50 mM Tris-HCl [pH7.5], 150 mM NaCl, 0.1 % SDS, 1 % Triton X-100, and 1 % sodium deoxycholate) containing phenylmethanesulfonyl fluoride, sodium fluoride, sodium orthovanadate, and protease inhibitor cocktail. The homogenates were sonicated on ice, incubated for 30 min, and centrifuged (16 000×*g*, 4 °C, 10 min). The protein content of supernatants was determined using the BCA method using Pierce BCA Protein Assay kit (Thermo Fisher Scientific). Proteins were mixed with loading buffer (54.1 % glycerol, 0.05 % bromophenol blue, 158.9 mM Tris-HCl [pH 6.8], and 4.76 % SDS), and protein samples (20 μg) were electrophoresed using SDS-PAGE. Next, protein samples were transferred to a PVDF membrane (0.45 μm or 0.20 μm; Millipore, Burlington, MA, USA) and blots were blocked by Blocking One (Nacalai Tesque, Kyoto, Japan). Membranes were incubated overnight at 4 °C with the following primary antibodies: IBA-1 (1:1000; Wako) and GAPDH (1:4000; MBL, Tokyo, Japan). Thereafter, the membranes were incubated with appropriate secondary antibodies for 1 h at room temperature. Each antibody was diluted in Can Get Signal solution (TOYOBO). EzWest Lumi Plus (ATTO, Tokyo, Japan) was used as the detection reagent. Blot images were digitally captured using a ChemiDoc MP imager (Bio-Rad), and luminescence intensities were calculated using ImageLab (Bio-Rad), corrected for GAPDH levels, and evaluated as a ratio to the sham group.

### 2.24 Statistical analysis

Data are expressed as the means ± standard error of mean (SEM) for each group. Statistical significance was evaluated using Student's *t*-test and one-way or two-way analysis of variance (ANOVA), followed by Dunnett's or Tukey's multiple comparison tests. *P* < 0.05 was considered statistically significant. Statistical analyses were performed using GraphPad version 9.0 (GraphPad Software, San Diego, CA, USA).

## 3 Results

### 3.1 NBD-TEEPO: A screening probe for lipid radical detection

The most effective approach to search for lipid radical inhibitors is to use competitive reactions between detection probes and library compounds that target lipid radicals. Two key points must be considered to ensure reliable results. First, the data should not provide false negative results (fluorescence emission) upon direct reactions between the probe and library compounds. Second, the reactivity between the detection probe and the lipid radicals must be sufficiently rapid. With these considerations in mind, we initially developed three profluorescent nitroxide probes with different α-positioning groups, including α-substituted nitroxide TEEPO, which does not react with ascorbic acid [26]; 2,2,6,6-tetramethyl-4-(4-nitrobenzo [1,2,5]oxadiazol-7-ylamino)piperidine-1-oxyl (NBD-TEMPO), NBD-Pen, and NBD-TEEPO. We evaluated their reactivities with seven reductants, including typical antioxidants and lipid radical inhibitors (Figs. S1A and B). NBD-TEMPO reacted with several antioxidants (50 μM) in the presence of liposomes, and NBD-Pen showed a slight reaction with ascorbic acid, vitamin E, and edaravone (sold under the brand name Radicava), resulting in a slight increase in the fluorescence intensity. By contrast, NBD-TEEPO did not react with any of the antioxidants.

The reactivities of these fluorescent probes toward carbon-centered radicals were evaluated based on the heat of formation (Δ*H*), calculated using DFT calculations. The negative Δ*H* values were −41.7 (TEMPO), −38.3 (TEEPO), and −38.2 (Pen) kcal mol^−1^, indicating that all three probes are comparable (Fig. S2). In fact, the three probes showed comparable rates of fluorescence increase in the liposome system using AAPH as a lipid peroxidation initiator (Fig. S3A). While the inhibitory activity of nitroxides against lipid peroxidation depends on their lipophilicity, clogP values of NBD-Pen and NBD-TEEPO are comparable and higher than that of NBD-TEMPO [15]. These findings suggest that competitive reactions between NBD-TEEPO and library compounds against lipid radicals can be used to search for highly active lipid radical inhibitors more effectively and conveniently.

Therefore, we chose NBD-TEEPO as the profluorescent probe and assessed its reactivity toward lipid-derived radicals using Fe^2+^ in addition to AAPH in the liposome system. The fluorescence intensity of NBD-TEEPO did not change in the absence of lipid radical initiators and increased in a concentration- and time-dependent manner in the presence of initiators (Figs. S3B and C). Moreover, this increase diminished upon the addition of edaravone, a medication employed in the treatment of stroke and amyotrophic lateral sclerosis, which was previously developed as a lipid radical scavenger. Finally, LC/MS/MS analysis showed that NBD-TEEPO reacted and formed adducts with arachidonic acid–derived radicals (Fig. S3D). These findings affirm that 1) NBD-TEEPO reacts with lipid radicals generated within liposomes, and 2) competitive reactions with library compounds can efficiently facilitate the search for lipid radical inhibitors because of the rapid reaction rate constant of the reaction between probe and lipid radicals.

### 3.2 Compound screening using NBD-TEEPO in liposomes

**Fig. 1:**
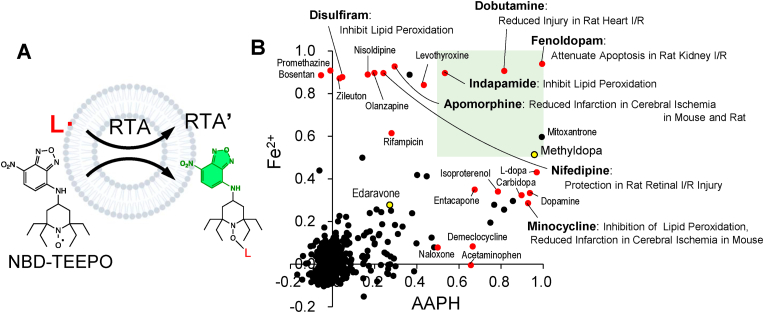
Lipid radical scavenger screening using a fluorescent probe in a liposome system. (**A**) NBD-TEEPO reacts with lipid radicals (L･) and turns fluorescent. RTAs compete with NBD-TEEPO and scavenge lipid radicals. (**B**) Inhibition rates of the compounds against two initiators (AAPH and Fe^2+^) are plotted. Beneficial properties of compounds with inhibitory activity against AAPH or Fe^2+^ greater than 0.5 were searched in the PubMed database. Red circles indicate compounds with reported protective activity against diseases related to oxidative stress. Edaravone and methyldopa are represented as yellow circles. The green square shows the region where both AAPH and Fe^2+^ values are greater than 0.5. See also Table 1. (For interpretation of the references to color in this figure legend, the reader is referred to the Web version of this article.)

Based on these findings, we constructed a compound screening system using NBD-TEEPO with liposomes and employing AAPH and Fe^2+^ as stimulants (Fig. 1A) to search for lipid radical inhibitors. We evaluated 766 compounds from the FDA-approved drug library with 384-well plates. Several compounds exhibited high lipid radical inhibitory activity (Fig. 1B). All test compounds, except idarubicin, did not directly react with NBD-TEEPO, and the reaction between idarubicin and NBD-TEEPO was negligible, thus eliminating the possibility of a false negative result (Figs. S3E and F). Notably, among hit compounds, we listed reported lipid peroxidation inhibitors, such as disulfiram [27], minocycline [28], and indapamide [29]. Consequently, we designated compounds with activity values of 0.5 or higher as hit compounds and conducted a PubMed search for reports on their inhibitory effects against ischemia-reperfusion injury, cerebral infarction, Alzheimer's disease, and other diseases thought to be associated with oxidative stress. In addition, we compiled a list of their molecular weights and octanol/water distribution coefficient (logP) values (Table 1). Notably, dobutamine, fenoldopam, and nifedipine, which exhibited high activity in this screening system, have been reported to exert protective effects in ischemia-reperfusion models of the heart, kidney, and retina. In addition, apomorphine and minocycline were identified as repurposed compounds with protective effects in a cerebral infarction model.

**Table 1:** Screening hit compounds with reported protective activity against diseases related to oxidative stress.

Compound Name	AAPH	Fe^2+^	Target Disease/Usage	Function	Disease model	Proposed Mechanism	PubMed ID	MW	logP
Fenoldopam	1.00	0.94	Hypertension	Dopamine D1-receptor agonist	Renal ischemia-reperfusion	Attenuation of NFκB signaling	16771250	305.8	2
					Acute kidney injury after cardiac surgery	Renal vasodilatation, inhibition of tubular sodium reabsorption	18068064		
Apomorphine	0.30	0.93	Parkinson disease	Dopamine agonist	MCAO	No mention	29220863	267.3	2
					Myocardial reperfusion	Chelation of Cu^2+^ and Fe^2+^	12911268		
					Alzheimer disease (3xTg)	Antioxidation, enhancement of Aβ degradation	21387370		
Promethazine	−0.01	0.91	Antiallergic	Histamine receptor H1 antagonist	Renal ischemia-reperfusion	Unknown	19686533	284.4	4.81
					MCAO	Inhibition of mitochondrial permeability transition	15263028		
					Light-induced retinal degeneration	Inhibition of lipid peroxidation	4423476		
Dobutamine	0.82	0.91	Cardiotonic	β1-Adrenergic receptor agonist	Myocardial ischemia-reperfusion	HO-1 induction	23531454	301.4	3.6
Olanzapine	0.20	0.90	Schizophrenia	MARTA	MCAO	No mention	17113959	312.4	4.094
					Cerulein-induced pancreatitis	Inhibition of ferroptosis	34562639		
					Retinal neuronal ischemia		23215752		
Indapamide	0.53	0.90	Hypertension	Thiazide related diuretic	Myocardial ischemia-reperfusion	Free-radical scavenging	7508061	365.8	2.2
Nifedipine	0.24	0.90	Antihypertensive	Calcium channel L type blocker	Bilateral carotid ligation-induced cerebral ischemia	Inhibition of lipid peroxidation	1791915	346.3	2.2
					Ischemic retinal dysfunction	Inhibition of Ca^2+^ overload	2097313		
					Cuprizone-induced demyelination	Modulating NF-κB and Nrf2 signaling	33709265		
Nisoldipine	0.17	0.89	Antihypertensive	Calcium channel L type blocker	Ischemic renal injury	Improving hemodynamics	4057426	388.4	3.26
					Myocardial infarction	Unknown	2581092		
Rifapentine	0.37	0.89	Tuberculosis	Nucleic acid synthesis inhibitor	No report			877.0	4
Bosentan	−0.05	0.88	Pulmonary arterial hypertension	Endothelin receptor antagonist	Ischemia/reperfusion-induced injury in ovaries	Blocking endothelin receptors	23880599	551.6	3.7
					Ischemia/reperfusion injury in skeletal muscle	Blocking endothelin receptors	11470261		
					Myocardial ischemia-reperfusion	Blocking endothelin receptors	16335785		
					Diabetic retinopathy (db/db)	Blocking endothelin receptors	30428543		
					Hypertensive retinopathy	Blocking endothelin receptors	19829016		
					Glaucoma (DBA/2J)	Blocking endothelin receptors	25132557		
					Alzheimer disease (Tg2576)	Blocking endothelin receptors	15894408		
Disulfiram	0.05	0.88	Antialcohol dependence	Aldehyde dehydrogenase inhibitor	Ischemia-induced ovary damage	Inhibition of lipid peroxidation	21756070	296.5	3.88
					Liver ischemia-reperfusion	Scavenger	6516872		
					Renal ischemia-reperfusion	Inhibition of pyroptosis	35837696		
					Retinitis pigmentosa (rd10)	Inhibition of retinoic acid synthesis	35302843		
					Diabetic retinopathy	Inhibition of iNOS	32993012		
					Alzheimer disease (5xFAD)	Enhancement of ADAM10 expression	29358714		
Zileuton	0.03	0.87	Asthma	5-lipoxygenase inhibitor	MCAO	Inhibition of 5-lipoxygenase	19309543	236.3	0.9
					Renal ischemia-reperfusion	Inhibition of 5-lipoxygenase	15266012		
					NaIO_3_ model (AMD/RP model)	Inhibition of 5-lipoxygenase and lipid peroxidation	35251467		
					Laser-induced CNV	Inhibition of LTB4	30232269		
					Alzheimer disease (Tg2576)	Inhibition of 5-lipoxygenase	21435457		
Levothyroxine	0.43	0.84	Thyroid hormone replenisher	Thyroid hormone thyroxine (T4)	Hepatic ischemia-reperfusion	No mention	17187421	776.9	4
					Myocardial ischemia-reperfusion	No mention	30200092		
					Alzheimer disease (STZ rat)	No mention	29927658		
Rifampicin	0.28	0.61	Tuberculosis	RNA polymerase inhibitor	MCAO	Hydroxyl radical scavenger	14670633	822.9	2.7
					APAP-induced liver injury	Activation of AMPK-Nrf2	31678598		
Mitoxantrone	0.99	0.60	Antineoplastic	Topoisomerase II inhibitor	No report			444.5	−3.1
Methyldopa	0.96	0.51	Hypertension	α2-Adrenergic receptor agonist	Spontaneously Hypertensive Pregnant	No mention		211.2	−1.79
L-dopa	0.97	0.43	Parkinson disease	Dopamine precursor	Diabetic retinopathy	Restoration of dopamine level	24431431	197.2	0.05
					Cerebral ischemia-reperfusion		33328857		
					Alzheimer disease (TgCRND8)	Stimulation of the dopaminergic system	18079024		
Entacapone	0.68	0.35	Parkinson disease	COMT inhibitor	Renal ischemia-reperfusion	Scavenging peroxynitrite	33675481	305.3	2.8
					Renal ischemia-reperfusion	Inhibition of ferroptosis	35691001		
Isoproterenol	0.79	0.34	Antivertigo, Cardiotonic, Bronchodilator	β-Adrenergic receptor agonist	Ischemia-reperfusion lung injury		15916984	211.3	1.4
					Retinopathy of prematurity	Decreasing VEGF expression	22410551		
					Diabetic retinopathy	Blocking TNFα production	20493839		
Dopamine	0.94	0.33	Myocardial infarction	Dopamine receptor agonist	Ischemic renal injury	Anti-inflammation	15172883	153.2	−0.98
Carbidopa	0.90	0.32	Parkinson disease	Aromatic l-amino acid decarboxylase inhibitor	Renal ischemia-reperfusion	Inhibition of 5-HT synthesis	34599937	226.2	−1.9
					MCAO		34035485		
Norepinephrine	0.86	0.30	Myocardial infarction	Adrenergic receptor agonist	No report			169.2	−1.24
Minocycline	0.93	0.29	Antibacterial	Inhibit 30S ribosome	MCAO	Inhibition of oxidative stress	15862784	457.5	0.05
					Retinal ischemia-reperfusion	Anti-inflammation and antioxidation	34295142		
					Light-induced retinal degeneration	Anti-inflammation	26576678		
					Laser-induced CNV	Microglia inhibitor	23977149		
					Diabetic retinopathy	Anti-inflammation	15855346		
					Endotoxin-induced uveitis	Inactivating microglia	31354229		
					Retinal neuronal ischemia	Antioxidation	16051195		
Epinephrine	0.77	0.28	Anaphylaxis	Adrenergic receptor agonist	No report			183.2	−1.37
Tigecycline	0.81	0.26	Antibacterial	Inhibit 30S ribosome	No report			585.7	0.8
Nalbuphine	0.75	0.19	Analgesic	Opioid receptor agonist	No report			357.4	1.4
Demeclocycline	0.67	0.08	Antibacterial	Inhibit 30S ribosome	MCAO	Inhibition of carpain	16091365	464.9	0.2
Naloxone	0.50	0.08	Narcotic antagonist	Opioid receptor antagonist	Cerebral ischemia-reperfusion		11338200	327.4	2.09
					Renal ischemia-reperfusion	No mention	23523991		
					Myocardial ischemia-reperfusion	Inhibition of *p*-JNK expression	24634599		
					Retinal ischemia	No mention	8083567		
Acetaminophen	0.66	−0.01	Anti-inflammatory		Myocardial ischemia-reperfusion	Antioxidation	11356619	151.2	0.46
					Gastric mucosal injury by ischemia-reperfusion	Antioxidation	9211566		
					Rhabdomyolysis-induced renal injury	Antioxidation	20133658		

We have also reported that rifampicin and promethazine, identified as ferroptosis inhibitors in acute kidney injury [30], display marked inhibitory effects on lipid radicals. To date, the search for compounds with antioxidant properties has relied on the selection of candidate drugs based on the expertise of researchers, chemical modification of compounds with known antioxidant properties, and screening systems developed using conventional methods. However, our newly developed compound screening system, which relies on competitive reactions between fluorescent probes and library compounds with lipid radicals with exceptionally fast reaction rates, allows for relatively simple and unbiased identification of potential candidate compounds. Multiple studies have demonstrated the protective effects of drugs with inherently different pharmacological effects in animal disease models (Table 1). This objective evidence strongly supports the hypothesis that these screening hit compounds possess lipid radical inhibitory capability and underscores the significant involvement of lipid radicals in the onset and progression of selected diseases.

### 3.3 Application 1 of screening hit compounds: light-induced retinal damage model

To validate our methodology, we investigated whether the hit compounds identified by screening exerted inhibitory effects on disease progression in animal models. We selected the retina as the target organ because of its abundance of PUFAs, particularly docosahexaenoic acid [31], and high oxygen consumption [31,32], which makes it susceptible to lipid peroxidation. Accumulation of lipid peroxidation products has been observed in the retinas of patients with dry-type age-related macular diseases [33] as well as in animal models of light-induced retinal damage [34]. In addition, increased oxidative stress was monitored in a retinal injury model using a profluorescent nitroxide probe [[35], [36], [37]].

**Fig. 2:**
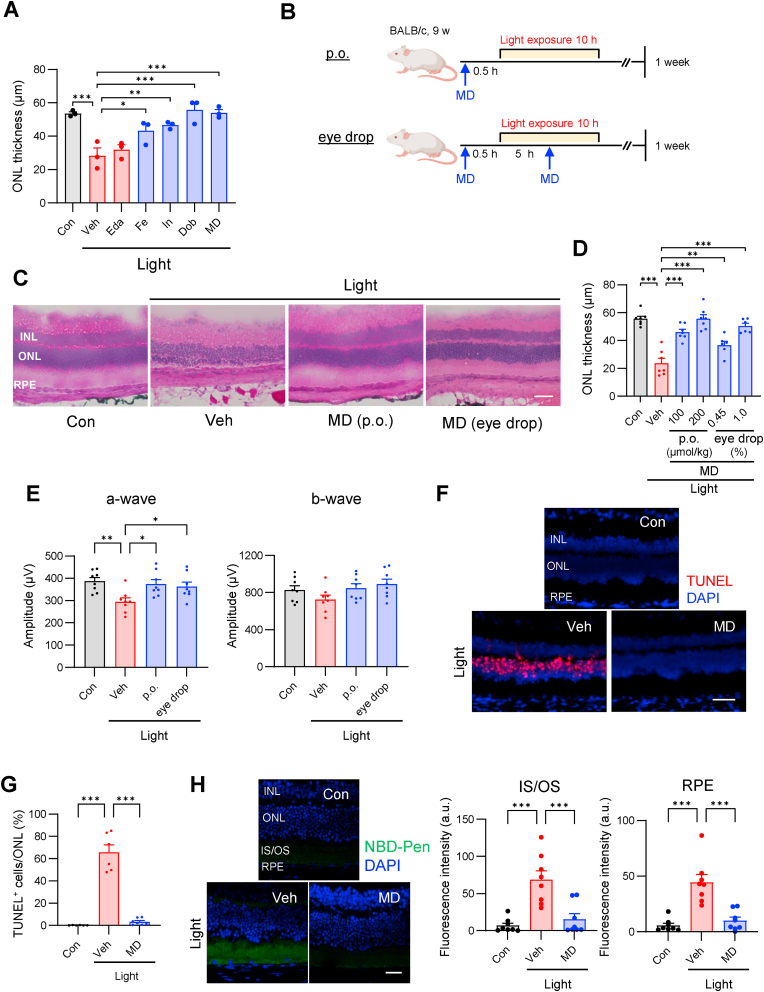
Screening hit compounds attenuate light-induced retinal degeneration. (**A**) Edaravone (Eda), fenoldopam (Fe), indapamide (In), dobutamine (Dob), or methyldopa (MD) were administered intraperitoneally (5.0 μmol/kg) 30 min before light exposure. *n* = 3 mice. (**B**) MD was administered orally (200 μmol/kg, p.o.) 30 min before light exposure (C to H) or using eye drops (1 % MD) 30 min before and 5 h after the start of light exposure (C and E). (**C**) HE staining of retinas from mice 7 d after the end of light exposure. (**D**) ONL thickness was measured in (C). *n* = 7 mice, except *n* = 6 mice for eye drop administration. (**E**) Electroretinographic evaluation of a- and b-wave amplitudes. *n* = 8 mice. (**F** and **G**) Retinal cell death was evaluated using TUNEL staining 24 h after the end of light exposure. *n* = 6, 7, 8 mice from left to right. (**H**) NBD-Pen (5.0 μmol/kg) was administered intraperitoneally at the end of light exposure. After 30 min, the eyes were enucleated. *n* = 8 mice. Scale bar, 50 μm. All data are presented as the means ± SEM. *p < 0.05, **p < 0.01, ***p < 0.001 by one-way ANOVA followed by Dunnett's multiple comparison tests.

In this study, based on results obtained using the FDA library, we selected approved drugs (fenoldopam, indapamide, dobutamine, mitoxantrone, and MD) with activity values above 0.5 (Fig. 1B) and evaluated their effects in a light-induced retinal damage model. Mitoxantrone, a topoisomerase inhibitor, was excluded owing to concerns regarding its toxicity. When administered intraperitoneally at the same dose, the other four drugs showed protective effects against retinal damage (Fig. 2A). In contrast, edaravone, which is reported to be effective in this model [38], showed no inhibitory effects at the low dose used in our study (50 μmol/kg).

Despite their different pharmacological mechanisms of action, all the four drugs showed similar protective effects against retinal damage. This further supports the notion that lipid radicals are appropriate targets in animal models of light-induced retinal damage and highlights the efficacy of our screening system.

### 3.4 Retina-protective effects of MD

MD was selected as a potential repurposed compound from the four drugs evaluated. We selected MD because of its relatively wide safety margin at clinical dosages and the absence of reported adverse effects on the fundus or skin [39]. We observed that both oral and eye drop administration of MD led to dose-dependent inhibition of the reduction in the ONL thickness (Fig. 2B–D, Figs. S4A and B). Additionally, MD significantly inhibited the reduction in the electroretinogram a-wave, a measure of retinal function (Fig. 2E and Fig. S4C) and decreased the number of TUNEL-positive cells during retinal damage (Fig. 2F and G). Furthermore, MD effectively suppressed the decrease in ONL thickness and protected against retinal damage in an MNU-treated animal model (Fig. S5).

The blood–retinal barrier (BRB) blocks the entry of harmful substances from the blood into retinal tissue. Therefore, we confirmed whether the orally administered MD migrated to the retina (Figs. S6A and B). MD is a substrate of l-amino acid transporter (LAT), which is expressed in the BRB, cornea, and intestinal wall [40]. Consequently, treatment of mice with 2-aminobicyclo[2.2.1]heptane-2-carboxylic acid (BCH), a LAT inhibitor, significantly reduced the migration of MD to the retina (Fig. S6C) and attenuated its protective effect against retinal damage (Fig. S6D). Furthermore, MD is metabolized to methyldopamine, which acts as an α2 receptor agonist. Clonidine and brimonidine, which are α2 receptor agonists, have been reported to suppress retinal damage [41,42]. Therefore, we investigated whether the retinoprotective effect of MD depended on α2 receptors and found that pre-administration of yohimbine, an α2 receptor blocker, did not affect the retinoprotective effect of MD (Fig. S6E). Moreover, MD significantly inhibited lipid radical generation in the inner and outer segments as well as in the RPE of the light-induced retinal damage mouse model (Fig. 2H). Additionally, MD treatment abolished the accumulation of oxidized phosphatidylcholine recognized by the E06 antibody and acrolein-modified proteins, which are lipid peroxidation end products (Figs. S6F and G).

These findings highlight the practical efficacy of MD against light- or MNU-induced retinal damage. Furthermore, the generation of lipid radicals may be a key factor in the pathogenesis and progression of acute retinal disease in animal models.

### 3.5 MD inhibits complement system activation

**Fig. 3:**
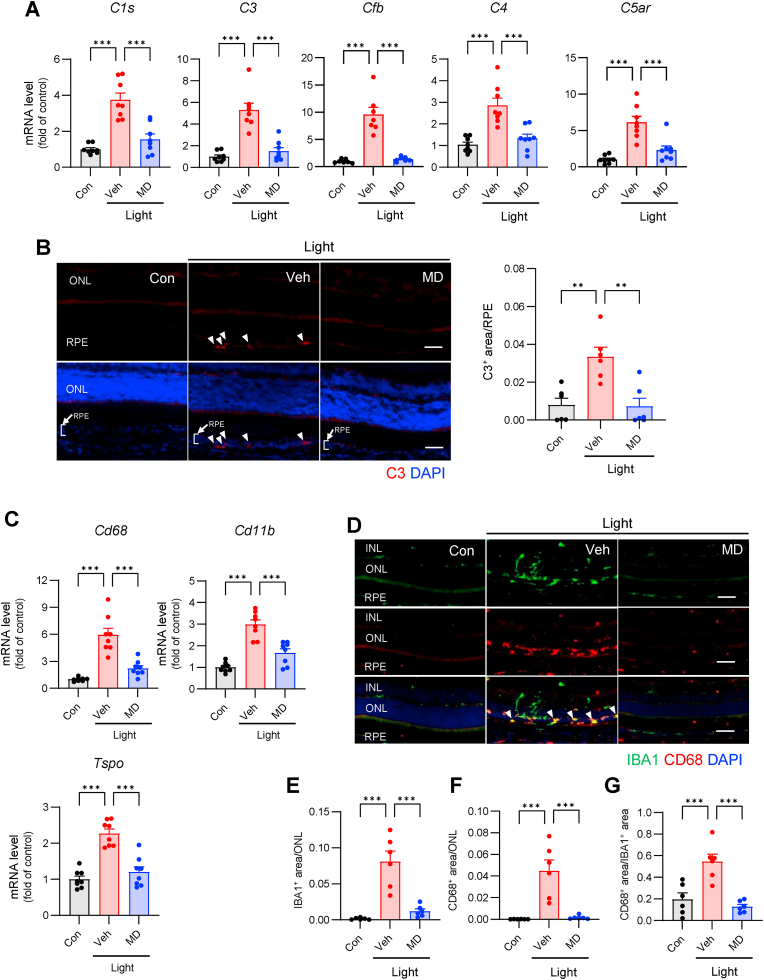
Methyldopa inhibits complement and glial cell activation. Methyldopa (200 μmol/kg) was administered orally 30 min before light exposure. (**A**) mRNA expression levels of complement-related genes in the retina were evaluated 24 h after the end of light exposure. *n* = 8 mice, except *n* = 7 mice for *Cfb*. (**B**) Representative fluorescence images with statistical comparisons between the indicated groups. C3 protein was stained 2 d after the end of light exposure. Arrowheads indicate the C3^+^ area. Scale bar, 50 μm. *n* = 6 mice. (**C**) Expression levels of the activated MG/MΦ marker genes in the retina were evaluated 24 h after the end of light exposure. *n* = 8 mice. Representative fluorescence images with statistical comparisons between the indicated groups. (**D** to **G**) Four days after light exposure, the eyes were enucleated. Arrowheads indicate CD68/IBA1 double-positive activated MG/MΦ. The average area of IBA1^+^ (E) and CD68^+^ (F) cells in ONL and the ratio of CD68^+^ area per IBA1^+^ cells (G) are shown. *n* = 6 mice. Scale bar, 50 μm. All data are presented as the means ± SEM. **p < 0.01, ***p < 0.001 by one-way ANOVA followed by Dunnett's multiple comparison tests.

Lipid peroxidation products are known to activate retinal myeloid cells, including the complement system and MG/MΦ, which play crucial roles in the pathological progression of dry age-related macular degeneration (d-AMD) [43]. Complement activation is essential for the pathogenesis and progression of d-AMD [44]. Similarly, complement activation has been observed in a light-induced retinal damage model [45], and protective effects against retinal damage have been reported in complement receptor-knockout mice [46]. Therefore, we investigated the effectiveness of MD in preventing complement activation in a mouse model of light-induced retinal damage. MD treatment effectively suppressed the elevated mRNA expression of complement factors (*C1s*, *C3*, *Cfb*, *C4*, and *C5ar*) in light-induced retinal damage (Fig. 3A). Furthermore, pre-administration of MD inhibited the accumulation of C3 protein in the upper part of the RPE (Fig. 3B). Additionally, MD significantly reduced the elevated expression of complement factors in complement-producing MG/MΦ and RPE cells (Fig. S7).

The mRNA levels of *Cd68*, *Cd11b*, and *Tspo*, which serve as activation markers for MG/MΦ, were elevated in the mouse model of light-induced retinal damage. Treatment with MD effectively suppressed the expression of these genes (Fig. 3C). Moreover, we observed a significant migration of IBA1-positive MG/MΦ to the damaged ONL in the damaged group, which co-localized with CD68, an activation marker (Fig. 3D–G). In contrast, the MD treatment group showed minimal migration of MG/MΦ to the ONL and reduced activation. Additionally, MD treatment abolished the increased mRNA expression of the activation markers *Gfap*, *Vim*, and *Slc1a3* in Müller cells, retina-specific glial cells (Fig. S8A). MD suppressed the elevated expression of the GFAP protein (Fig. S8B).

These results indicated that MD effectively suppressed the activation of the complement system and glial cells, which is crucial for the development of pathological conditions in animal models of light-induced retinal damage. Our findings suggested that lipid radicals are necessary for disease induction in this animal model of acute retinal injury.

### 3.6 Application 2 of screening hit compounds: BCAS model

**Fig. 4:**
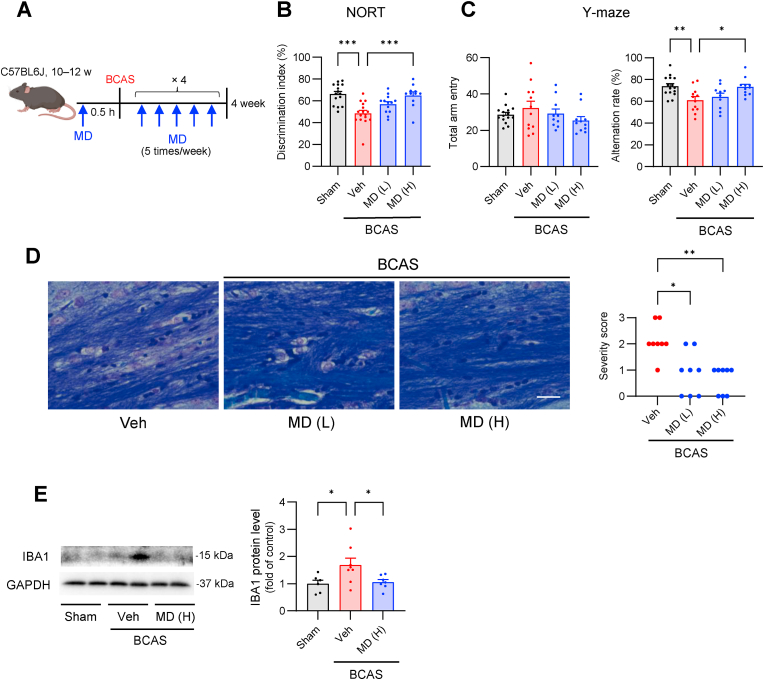
Methyldopa protects against pathophysiology of vascular dementia. (**A**) MD was administered orally 30 min before BCAS surgery and five times weekly after surgery: MD (L), 88.7 μmol/kg; MD (H), 177.3 μmol/kg. (**B**) Exploratory preference for the novel object in the test session, 4 weeks after BCAS surgery. *n* = 14, 15, 13, 11 mice from left to right. (**C**) Total arm entry and alternation rate in the Y-maze test, 4 weeks after BCAS surgery. *n* = 15, 12, 10, 11 mice from left to right. (**D**) Klüver-Barrera staining for histological evaluation. The corpus callosum of BCAS mice was sectioned 4 weeks after surgery. The severity of the white matter lesion in the corpus callosum was scored. Scale bar, 25 μm. *n* = 8 mice. (E) Expression of IBA1 protein in the corpus callosum was evaluated 1 week after BCAS surgery. *n* = 6, 8, 7 mice from left to right. All data are presented as the means ± SEM. *p < 0.05, **p < 0.01, ***p < 0.001 by one-way ANOVA followed by Dunnett's multiple comparison tests.

Next, we investigated the protective effects of MD against chronic diseases. The top screened compounds showed protective effects in various animal models of cerebrovascular disorders (Table 1). Similar to the retinal tissue, the brain is rich in PUFAs [47], and lipid peroxidation products accumulate in the plasma and brain of patients with vascular dementia and early cognitive dysfunction [48]. Therefore, we examined the effects of MD on cognitive dysfunction using a chronic cerebral hypoperfusion model, BCAS, which mimics subcortical ischemic vascular dementia (Fig. 4A) [25].

To assess working memory impairment resulting from chronic hypoperfusion, we used the NORT, a measure of non-spatial working memory [49]. Four weeks after the occurrence of BCAS, MD treatment significantly prevented the decrease in the percentage of novel object recognition (Fig. 4B). Additionally, spatial working memory was evaluated via the Y-maze test. The number of arm entries did not differ between the two groups (Fig. 4C). The percentage of successful arm changes decreased 4 weeks after BCAS occurrence but was preserved by MD administration (Fig. 4C). Notably, MD administration did not alter CBF in the BCAS mouse model (Fig. S9).

Furthermore, MD administration attenuated white matter injury observed 4 weeks after BCAS occurrence (Fig. 4D). Additionally, MD markedly reduced the expression levels of a microglial marker (IBA1), which were elevated in the BCAS model animals (Fig. 4E).

These findings demonstrated that MD, a screening hit compound, effectively ameliorated memory and white matter deficits in BCAS model animals, representing a model of chronic memory impairment.

## 4 Discussion

In this study, we developed a novel high-throughput screening technique targeting lipid radicals that allowed the identification of compounds with high lipid radical inhibitory activity. This technique offers specificity, reliability, reproducibility, and efficiency for the assessment of inhibitory activity. Importantly, we were able to identify repurposed compounds that have already been reported. Among the four drugs with different mechanisms of action, we observed similar protective effects in animal models of light-induced retinal damage. Focusing on MD, we propose the involvement of lipid radicals in the development and progression of diseases in animals with retinal damage and in BCAS model mice.

Despite the involvement of oxidized lipids in the pathogenesis of various diseases, identification of appropriate drug candidates remains limited. In this study, we developed a new screening system that targets lipid radicals, which are critical molecules in the chain reactions of lipid peroxidation. Nitroxide, a stable organic spin compound used as a screening probe in this system, shows extremely high reactivity (k = 10^7−9^ M^−1^s^−1^) toward carbon-centered free radical species [13]. Indeed, in our study, the probe demonstrated a high bond formation energy and diffusion-limited kinetics with carbon-centered radicals, enabling effective screening based on competitive reactions (Fig. S2).

In our experimental setup, we stimulated liposomes containing EggPC with Fe^2+^ or AAPH to generate lipid radicals. Initially, we anticipated that the candidate inhibitors, identified by screening, would be the same for lipid radicals generated by Fe^2+^ or AAPH stimulation, because the reactions between lipid radicals and compounds involve radical or proton transfer reactions. In other words, we expected the inhibitory potencies for both stimuli to be comparable, resulting in a linear correlation between the activity values of the drugs for each stimulus. However, screening using FDA-approved compound libraries revealed that the types of drugs hit by Fe^2+^ or AAPH stimuli were markedly different. This finding suggests that, although lipid radicals are generally considered molecules generated through radical chain reactions, the types of lipid radical molecules generated differ depending on the presence or absence of iron, and the inhibitory effects of drugs against these radicals also differ considerably. Interestingly, ferrostatin-1 (activity: AAPH 0.20, Fe^2+^ 0.89) and liproxstatin-1 (activity: AAPH 0.28, Fe^2+^ 0.92), widely used as RTAs in ferroptosis research, showed significant inhibitory effects exclusively during iron stimulation. We previously found that the types of unsaturated fatty acid–derived lipid radicals [11] and oxidized phospholipids [50] can vary significantly depending on the stimulation, such as Fe^2+^ and/or AAPH. Although the reason for the different types of compounds showing inhibitory effects in the presence and absence of iron is unknown, this may indicate that the reactivity of lipid radicals with compounds varies greatly depending on their type. Utilizing drugs with different reactivities may help elucidate the diverse biological effects of lipid radicals with high reactivity.

By contrast, multiple compounds have shown effectiveness against the same disease. For instance, administration of promethazine, olanzapine, or rifampicin appears to reduce the onset and progression of stroke in a cerebral infarction model. These drugs exhibit different pharmacological mechanisms of action (Table 1). However, their ability to objectively inhibit lipid radicals demonstrates their involvement of lipid radicals in the pathogenic mechanisms in animal disease models. This screening system provides a new approach for identifying inhibitors of lipid radicals and oxidized lipids involved in diseases. This study provides new insights into the role of oxidized lipids in disease induction.

The FDA-approved compound MD, which was identified in our screening, acts as an α2 receptor agonist. In our study, oral or ophthalmic administration of MD reduced retinal damage in the animal models. Moreover, the migration of MD to the retina appears to be mediated by LAT, which is involved in amino acid transport. LAT1 shows broad substrate specificity and allows the passage of large neutral amino acids and amino acid-like drugs, such as MD, l-dopa, and melphalan [40].

MD inhibited retinal damage at doses of 100 and 200 μmol/kg (21.1 and 42.2 mg/kg) via oral administration and at a concentration of 0.45 and 1 % via ophthalmic administration in a dose-dependent manner. The oral LD_50_ of MD in mice is 5370 mg/kg (25 mmol/kg) [51]. The clinical dosage of MD used as a therapeutic agent for hypertension in adults ranges from 250 to 2000 mg/day [39]. The doses used in our study were lower than those used in clinical practice. Interestingly, l-dopa, a compound with a molecular structure similar to that of MD used in the treatment of Parkinson's disease, also showed a strong inhibitory effect on lipid radicals (Table 1). Three independent cohort analyses showed that patients prescribed l-dopa experienced a delay in the onset of AMD by approximately 8 years [52]. In addition, l-dopa treatment may reduce the number of anti-VEGF antibody injections required to treat wet-type AMD [53]. l-dopa is an anti-Parkinsonian drug contraindicated for closed-angle glaucoma. Conversely, MD is used to treat hypertension, and no adverse reactions to the eyes or skin have been reported, assuming ocular administration.

Recent studies have focused on oxidized lipid-induced cell death and ferroptosis. Moreover, oxidized lipids or complexes formed between oxidized products and proteins are involved in the induction of cytotoxicity and inflammatory responses beyond ferroptosis. For example, the oxidized lipid-protein complex carboxyethylpyrroles (CEPs) activate the complement system [54]. Recently, it was reported that CEP activates the complement system via Toll-like receptor 2 [55]. Furthermore, the subretinal administration of oxidized phospholipids induces angiogenesis [56]. The lipid whisker model proposes that unsaturated fatty acids in the plasma membrane increase their water solubility upon oxidation and protrude onto the surface of the plasma membrane where they act as ligands [57]. Oxidized lipids induce inflammatory responses via CD36 and SRB1 [58]. Conversely, in mice subjected to knockout of GPX4, an enzyme that scavenges oxidized lipids, retinal cell death occurs on postnatal day 21 leading to blindness [59]. Therefore, oxidized lipids may play a role in cellular damage and inflammatory responses. Furthermore, the effects of various oxidized lipids such as peroxides, aldehyde bodies, and protein complexes can vary considerably.

In this study, we targeted lipid radicals as critical molecules in the formation of oxidized lipids. However, the identification of specific oxidized lipids or subsequent signaling pathways responsible for disease onset remains limited. Lipid radicals and oxidized lipids are present in insufficient quantities, and they are diverse and highly reactive. In vivo detection and understanding of the effects of oxidized lipids in the body are currently limited. In such cases, using multiple inhibitors, as used in this study, may shed light on the involvement of lipid radicals and oxidized lipids in diseases and help elucidate disease mechanisms.

In this study, we proposed a reliable, reproducible, and high-throughput screening technique to identify lipid radical inhibitors, which are crucial molecules in lipid peroxidation. We also highlighted the protective effects of MD against retinal and brain diseases. The hit compounds against lipid radicals generated by Fe^2+^ or AAPH stimulation differed. This study emphasizes the need to reassess the involvement of oxidized lipids and lipid peroxidation in various diseases.

## Funding

This work was supported in part by an AMED-CREST grant (JP22gm0910013 to KY), JSPS KAKENHI grants (23H05481, 22H05572, 20H00493, and 18K19405 to KY), 10.13039/501100005927Daiichi Sankyo Foundation of Life Science, and 10.13039/501100008664Ono Medical Research Foundation to KY. This work was also supported by the Platform Project for Supporting Drug Discovery and Life Science Research of AMED.

## Data availability

All data needed to evaluate the conclusions in the paper are present in the paper and/or the Supplementary Materials.

## CRediT authorship contribution statement

**Ryota Mori:** Data curation, Formal analysis, Investigation, Methodology, Validation, Visualization, Writing – original draft, Writing – review & editing. **Masami Abe:** Data curation, Formal analysis, Investigation, Methodology, Validation, Visualization, Writing – original draft, Writing – review & editing. **Yuma Saimoto:** Data curation, Formal analysis, Investigation, Methodology, Validation, Visualization, Writing – original draft, Writing – review & editing. **Saki Shinto:** Data curation, Formal analysis, Investigation, Methodology. **Sara Jodai:** Data curation, Formal analysis, Validation, Visualization. **Manami Tomomatsu:** Data curation, Investigation. **Kaho Tazoe:** Investigation, Resources. **Minato Ishida:** Data curation, Formal analysis, Investigation. **Masataka Enoki:** Investigation. **Nao Kato:** Formal analysis, Investigation, Methodology. **Tomohiro Yamashita:** Resources. **Yuki Itabashi:** Investigation, Methodology. **Ikuo Nakanishi:** Investigation, Methodology, Software. **Kei Ohkubo:** Supervision, Writing – original draft. **Sachiko Kaidzu:** Formal analysis, Investigation, Methodology. **Masaki Tanito:** Supervision. **Yuta Matsuoka:** Methodology, Supervision. **Kazushi Morimoto:** Conceptualization, Project administration, Supervision, Writing – review & editing. **Ken-ichi Yamada:** Conceptualization, Methodology, Resources, Supervision, Writing – original draft, Writing – review & editing.

## Declaration of competing interest

The authors declare the following financial interests/personal relationships which may be considered as potential competing interests: Ken-ichi Yamada reports a relationship with FELIQS Corporation that includes: board membership, equity or stocks, and non-financial support. Ken-ichi Yamada holds patents for the screening system and for some of the compounds described herein. If there are other authors, they declare that they have no known competing financial interests or personal relationships that could have appeared to influence the work reported in this paper.

## References

[1] Imai Y., Kuba K., Neely G.G., Yaghubian-Malhami R., Perkmann T., van Loo G., Ermolaeva M., Veldhuizen R., Leung Y.H.C., Wang H., Liu H., Sun Y., Pasparakis M., Kopf M., Mech C., Bavari S., Peiris J.S.M., Slutsky A.S., Akira S., Hultqvist M., Holmdahl R., Nicholls J., Jiang C., Binder C.J., Penninger J.M. (2008). Identification of oxidative stress and toll-like receptor 4 signaling as a key pathway of acute lung injury. Cell.

[2] Di Gioia M., Spreafico R., Springstead J.R., Mendelson M.M., Joehanes R., Levy D., Zanoni I. (2019). Endogenous oxidized phospholipids reprogram cellular metabolism and boost hyperinflammation. Nat. Immunol..

[3] West X.Z., Malinin N.L., Merkulova A.A., Tischenko M., Kerr B.A., Borden E.C., Podrez E.A., Salomon R.G., Byzova T.V. (2010). Oxidative stress induces angiogenesis by activating TLR2 with novel endogenous ligands. Nature.

[4] Dixon S.J., Lemberg K.M., Lamprecht M.R., Skouta R., Zaitsev E.M., Gleason C.E., Patel D.N., Bauer A.J., Cantley A.M., Yang W.S., Morrison B., Stockwell B.R. (2012). Ferroptosis: an iron-dependent form of nonapoptotic cell death. Cell.

[5] Zheng J., Conrad M. (2020). The metabolic underpinnings of ferroptosis. Cell Metabol..

[6] Huang D., Boxin O.U., Prior R.L. (2005). The chemistry behind antioxidant capacity assays. J. Agric. Food Chem..

[7] Shah R., Farmer L.A., Zilka O., Van Kessel A.T.M., Pratt D.A. (2019). Beyond DPPH: use of fluorescence-enabled inhibited autoxidation to predict oxidative cell death rescue. Cell Chem. Biol..

[8] Haidasz E.A., Van Kessel A.T.M., Pratt D.A. (2016). A continuous visible light spectrophotometric approach to accurately determine the reactivity of radical-trapping antioxidants. J. Org. Chem..

[9] Yamasaki T., Ito Y., Mito F., Kitagawa K., Matsuoka Y., Yamato M., Yamada K. (2011). Structural concept of nitroxide as a lipid peroxidation inhibitor. J. Org. Chem..

[10] Yamada K.I., Mito F., Matsuoka Y., Ide S., Shikimachi K., Fujiki A., Kusakabe D., Ishida Y., Enoki M., Tada A., Ariyoshi M., Yamasaki T., Yamato M. (2016). Fluorescence probes to detect lipid-derived radicals. Nat. Chem. Biol..

[11] Matsuoka Y., Izumi Y., Takahashi M., Bamba T., Yamada K.I. (2020). Method for structural determination of lipid-derived radicals. Anal. Chem..

[12] Yoshida Y., Shimakawa S., Itoh N., Niki E. (2003). Action of DCFH and BODIPY as a probe for radical oxidation in hydrophilic and lipophilic domain. Free Radic. Res..

[13] Bagryanskaya E.G., Marque S.R.A. (2014). Scavenging of organic C-centered radicals by nitroxides. Chem. Rev..

[14] Yamasaki T., Mito F., Ito Y., Pandian S., Kinoshita Y., Nakano K., Murugesan R., Sakai K., Utsumi H., Yamada K. (2011). Structure−Reactivity relationship of piperidine nitroxide: electrochemical, ESR and computational studies. J. Org. Chem..

[15] Yamasaki T., Ito Y., Mito F., Kitagawa K., Matsuoka Y., Yamato M., Yamada K. (2011). Structural concept of nitroxide as a lipid peroxidation inhibitor. J. Org. Chem..

[16] Frisch M.J. (2016).

[17] Becke A.D. (1993). Density‐functional thermochemistry. III. The role of exact exchange. J. Chem. Phys..

[18] Lee C., Yang W., Parr R.G. (1988). Development of the Colle-Salvetti correlation-energy formula into a functional of the electron density. Phys. Rev. B Condens. Matter.

[19] Hay P.J., Wadt W.R. (1985). Ab initio effective core potentials for molecular calculations. Potentials for K to Au including the outermost core orbitals. J. Chem. Phys..

[20] Keith T.A., Millam J.M. (2016).

[21] Bligh E.G., Dyer W.J. (1959). A rapid method of total lipid extraction and purification. Can. J. Biochem. Physiol..

[22] Enoki M., Shinto S., Matsuoka Y., Otsuka A., Kaidzu S., Tanito M., Shibata T., Uchida K., Ohira A., Yamato M., Yamada K.I. (2017). Lipid radicals cause light-induced retinal degeneration. Chem. Commun..

[23] Smith S.B., Yielding K.L. (1986). Retinal degeneration in the mouse. A model induced transplaceatally by methylnitrosourea. Exp. Eye Res..

[24] Shang P., Stepicheva N.A., Hose S., Zigler J.S., Sinha D. (2018). Primary cell cultures from the mouse retinal pigment epithelium. J. Vis. Exp..

[25] Shibata M., Ohtani R., Ihara M., Tomimoto H. (2004). White matter lesions and glial activation in a novel mouse model of chronic cerebral hypoperfusion. Stroke.

[26] Kinoshita Y., Yamada K., Yamasaki T., Mito F., Yamato M., Kosem N., Deguchi H., Shirahama C., Ito Y., Kitagawa K., Okukado N., Sakai K., Utsumi H. (2010). In vivo evaluation of novel nitroxyl radicals with reduction stability. Free Radic. Biol. Med..

[27] Misiorowski R.L., Chvapil M., Snider B.J., Weinstein P.R., Vostal J.J. (1983). Inhibition of lipid peroxidation in spinal cord homogenates by various drugs. Exp. Neurol..

[28] Morimoto N., Shimazawa M., Yamashima T., Nagai H., Hara H. (2005). Minocycline inhibits oxidative stress and decreases in vitro and in vivo ischemic neuronal damage. Brain Res..

[29] Uehara Y., Shirahase H., Nagata T., Ishimitsu T., Morishita S., Osumi S., Matsuoka H., Sugimoto T. (1990). Radical scavengers of indapamide in prostacyclin synthesis in rat smooth muscle cell. Hypertension.

[30] Mishima E., Sato E., Ito J., Yamada K.I., Suzuki C., Oikawa Y., Matsuhashi T., Kikuchi K., Toyohara T., Suzuki T., Ito S., Nakagawa K., Abe T. (2020). Drugs repurposed as antiferroptosis agents suppress organ damage, including AKI, by functioning as lipid peroxyl radical scavengers. J. Am. Soc. Nephrol..

[31] Fliesler A.J., Anderson R.E. (1983). Chemistry and metabolism of lipids in the vertebrate retina. Prog. Lipid Res..

[32] Ye X., Wang Y., Nathans J. (2010). The Norrin/Frizzled4 signaling pathway in retinal vascular development and disease. Trends Mol. Med..

[33] Suzuki M., Kamei M., Itabe H., Yoneda K., Bando H., Kume N., Tano Y. (2007). Oxidized phospholipids in the macula increase with age and in eyes with age-related macular degeneration. Mol. Vis..

[34] Sun M., Finnemann S.C., Febbraio M., Shan L., Annangudi S.P., Podrez E.A., Hoppe G., Darrow R., Organisciak D.T., Salomon R.G., Silverstein R.L., Hazen S.L. (2006). Light-induced oxidation of photoreceptor outer segment phospholipids generates ligands for CD36-mediated phagocytosis by retinal pigment epithelium: a potential mechanism for modulating outer segment phagocytosis under oxidant stress conditions. J. Biol. Chem..

[35] Rayner C.L., Bottle S.E., Martyn A.P., Barnett N.L. (2023). Preserving retinal structure and function with the novel nitroxide antioxidant. DCTEIO, Neurochem. Res..

[36] Rayner C.L., Gole G.A., Bottle S.E., Barnett N.L. (2014). Dynamic, in vivo, real-time detection of retinal oxidative status in a model of elevated intraocular pressure using a novel, reversibly responsive, profluorescent nitroxide probe. Exp. Eye Res..

[37] Rayner C.L., Bottle S.E., Gole G.A., Ward M.S., Barnett N.L. (2016). Real-time quantification of oxidative stress and the protective effect of nitroxide antioxidants. Neurochem. Int..

[38] Imai S., Inokuchi Y., Nakamura S., Tsuruma K., Shimazawa M., Hara H. (2010). Systemic administration of a free radical scavenger, edaravone, protects against light-induced photoreceptor degeneration in the mouse retina. Eur. J. Pharmacol..

[39] Whelton P.K., Carey R.M., Aronow W.S., Casey D.E., Collins K.J., Himmelfarb C.D., DePalma S.M., Gidding S., Jamerson K.A., Jones D.W., MacLaughlin E.J., Muntner P., Ovbiagele B., Smith S.C., Spencer C.C., Stafford R.S., Taler S.J., Thomas R.J., Williams K.A., Williamson J.D., Wright J.T., Levine G.N., O'Gara P.T., Halperin J.L., Past I., Al S.M., Beckman J.A., Birtcher K.K., Bozkurt B., Brindis R.G., Cigarroa J.E., Curtis L.H., Deswal A., Fleisher L.A., Gentile F., Goldberger Z.D., Hlatky M.A., Ikonomidis J., Joglar J.A., Mauri L., Pressler S.J., Riegel B., Wijeysundera D.N., Walsh M.N., Jacobovitz S., Oetgen W.J., Elma M.A., Scholtz A., Sheehan K.A., Abdullah A.R., Tahir N., Warner J.J., Brown N., Robertson R.M., Whitman G.R., Hundley J. (2017). ACC/AHA/AAPA/ABC/ACPM/AGS/APhA/ASH/ASPC/NMA/PCNA guideline for the prevention, detection, evaluation, and management of high blood pressure in adults: a report of the American college of cardiology/American heart association task force on clinical practice Guidelines. Hypertension.

[40] Puris E., Gynther M., Auriola S., Huttunen K.M. (2020). L-Type amino acid transporter 1 as a target for drug delivery. Pharm. Res. (N. Y.).

[41] Damico F.M., Gasparin F., Scolari M.R., Pedral L.S., Takahashi B.S. (2012). New approaches and potential treatments for dry age-related macular degeneration. Arq. Bras. Oftalmol..

[42] Chao H.M., Osborne N.N. (2001). Topically applied clonidine protects the rat retina from ischaemia/reperfusion by stimulating α2-adrenoceptors and not by an action on imidazoline receptors. Brain Res..

[43] Rathnasamy G., Foulds W.S., Ling E.A., Kaur C. (2019). Retinal microglia – a key player in healthy and diseased retina. Prog. Neurobiol..

[44] Armento A., Ueffing M., Clark S.J. (2021). The complement system in age-related macular degeneration. Cell. Mol. Life Sci..

[45] Rutar M., Natoli R., Albarracin R., Valter K., Provis J. (2012). 670-nm light treatment reduces complement propagation following retinal degeneration. J. Neuroinflammation.

[46] Song D., Sulewski M.E., Wang C., Song J., Bhuyan R., Sterling J., Clark E., Song W.C., Dunaief J.L. (2017). Complement C5a receptor knockout has diminished light-induced microglia/macrophage retinal migration. Mol. Vis..

[47] Svennerholm L. (1968). Distribution and fatty acid composition of phosphoglycerides in normal human brain. J. Lipid Res..

[48] Reed T., Perluigi M., Sultana R., Pierce W.M., Klein J.B., Turner D.M., Coccia R., Markesbery W.R., Butterfield D.A. (2008). Redox proteomic identification of 4-Hydroxy-2-nonenal-modified brain proteins in amnestic mild cognitive impairment: insight into the role of lipid peroxidation in the progression and pathogenesis of Alzheimer's disease. Neurobiol. Dis..

[49] Shibata M., Yamasaki N., Miyakawa T., Kalaria R.N., Fujita Y., Ohtani R., Ihara M., Takahashi R., Tomimoto H. (2007). Selective impairment of working memory in a mouse model of chronic cerebral hypoperfusion. Stroke.

[50] Matsuoka Y., Takahashi M., Sugiura Y., Izumi Y., Nishiyama K., Nishida M., Suematsu M., Bamba T., Yamada K. (2021). Structural library and visualization of endogenously oxidized phosphatidylcholines using mass spectrometry-based techniques. Nat. Commun..

[51] Toxicology and Carcinogenesis Studies of a-Methyldopa Sesquihydrate (CAS No. 41372-08-1) in F344/N Rats and B6C3F1Mice (Feed Studies), National Toxicology Program 348 (1989). 12704436

[52] Brilliant M.H., Vaziri K., Connor T.B., Schwartz S.G., Carroll J.J., McCarty C.A., Schrodi S.J., Hebbring S.J., Kishor K.S., Flynn H.W., Moshfeghi A.A., Moshfeghi D.M., Fini M.E., McKay B.S. (2016). Mining retrospective data for virtual prospective drug repurposing: L-DOPA and age-related macular degeneration. Am. J. Med..

[53] Figueroa A.G., Boyd B.M., Christensen C.A., Javid C.G., McKay B.S., Fagan T.C., Snyder R.W. (2021). Levodopa positively affects neovascular age-related macular degeneration. Am. J. Med..

[54] Hollyfield J.G., Perez V.L., Salomon R.G. (2010). A hapten generated from an oxidation fragment of docosahexaenoic acid is sufficient to initiate age-related macular degeneration. Mol. Neurobiol..

[55] Mulfaul K., Ozaki E., Fernando N., Brennan K., Chirco K.R., Connolly E., Greene C., Maminishkis A., Salomon R.G., Linetsky M., Natoli R., Mullins R.F., Campbell M., Doyle S.L. (2020). Toll-like receptor 2 facilitates oxidative damage-induced retinal degeneration. Cell Rep..

[56] Shaw P.X., Zhang L., Zhang M., Du H., Zhao L., Lee C., Grob S., Lim S.L., Hughes G., Lee J., Bedell M., Nelson M.H., Lu F., Krupa M., Luo J., Ouyang H., Tu Z., Su Z., Zhu J., Wei X., Feng Z., Duan Y., Yang Z., Ferreyra H., Bartsch D.U., Kozak I., Zhang L., Lin F., Sun H., Feng H., Zhang K. (2012). Complement factor H genotypes impact risk of age-related macular degeneration by interaction with oxidized phospholipids. Proc. Natl. Acad. Sci. U.S.A..

[57] Greenberg M.E., Li X.M., Gugiu B.G., Gu X., Qin J., Salomon R.G., Hazen S.L. (2008). The lipid whisker model of the structure of oxidized cell membranes. J. Biol. Chem..

[58] Salomon R.G., Gu X. (2011). Critical insights into cardiovascular disease from basic research on the oxidation of phospholipids: the γ-hydroxyalkenal phospholipid hypothesis. Chem. Res. Toxicol..

[59] Ueta T., Inoue T., Furukawa T., Tamaki Y., Nakagawa Y., Imai H., Yanagi Y. (2012). Glutathione peroxidase 4 is required for maturation of photoreceptor cells. J. Biol. Chem..

