# Supplementary material for: Construction of a screening system for lipid-derived radical inhibitors and validation of hit compounds to target retinal and cerebrovascular diseases
Source: Redox Biol. 2024 May 8;73:103186. doi: 10.1016/j.redox.2024.103186 (PMC11109892; doi:10.1016/j.redox.2024.103186)
Supplement: Multimedia component 1 [file mmc1.pptx]

## Slide 1
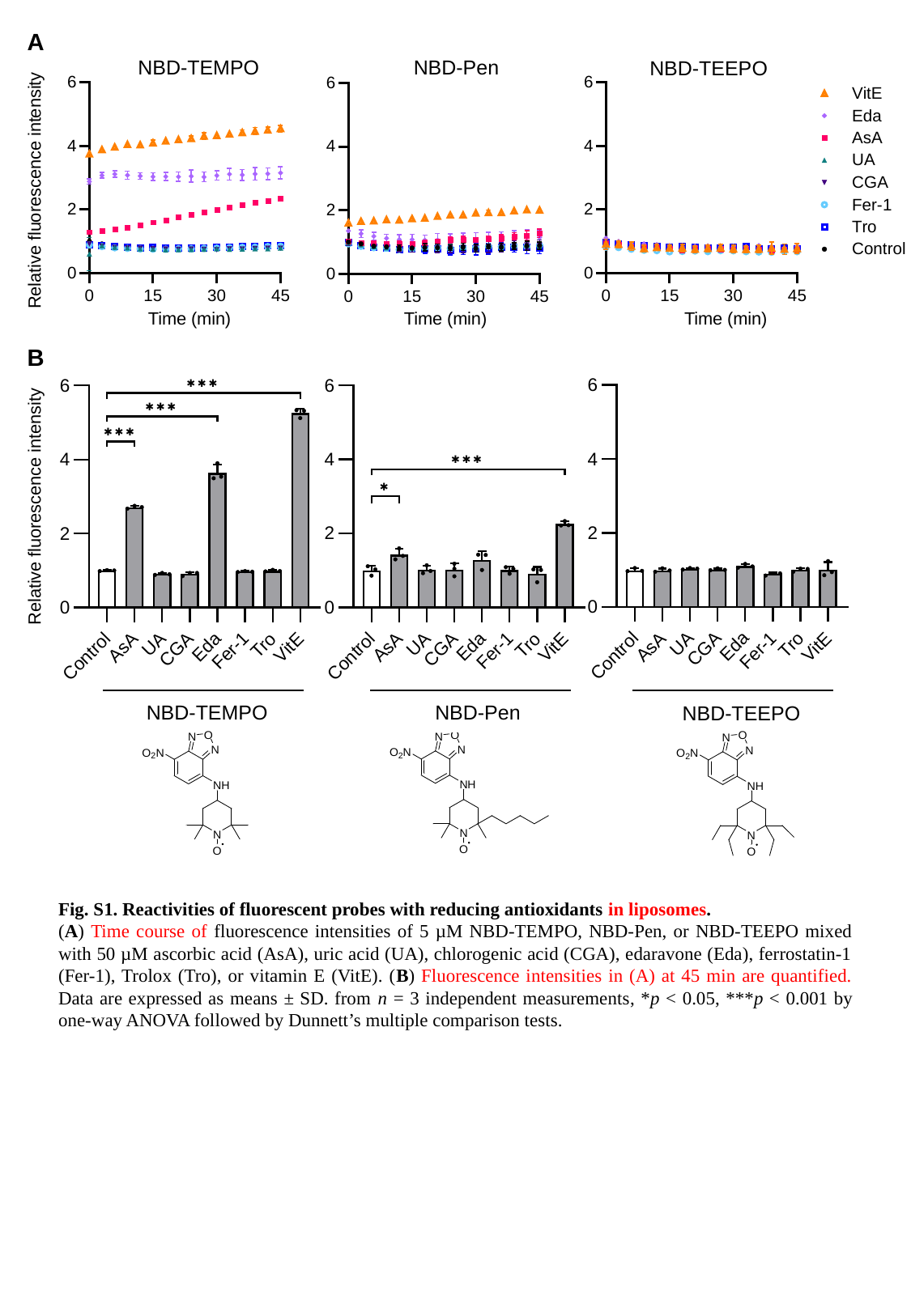

A
NBD-TEMPO
NBD-Pen
NBD-TEEPO
Relative fluorescence intensity
Time (min)
Time (min)
Time (min)
B
Relative fluorescence intensity
NBD-TEMPO
NBD-Pen
NBD-TEEPO
Fig. S1. Reactivities of fluorescent probes with reducing antioxidants in liposomes.
(A) Time course of fluorescence intensities of 5 µM NBD-TEMPO, NBD-Pen, or NBD-TEEPO mixed with 50 µM ascorbic acid (AsA), uric acid (UA), chlorogenic acid (CGA), edaravone (Eda), ferrostatin-1 (Fer-1), Trolox (Tro), or vitamin E (VitE). (B) Fluorescence intensities in (A) at 45 min are quantified. Data are expressed as means ± SD. from n = 3 independent measurements, *p < 0.05, ***p < 0.001 by one-way ANOVA followed by Dunnett’s multiple comparison tests.

## Slide 2
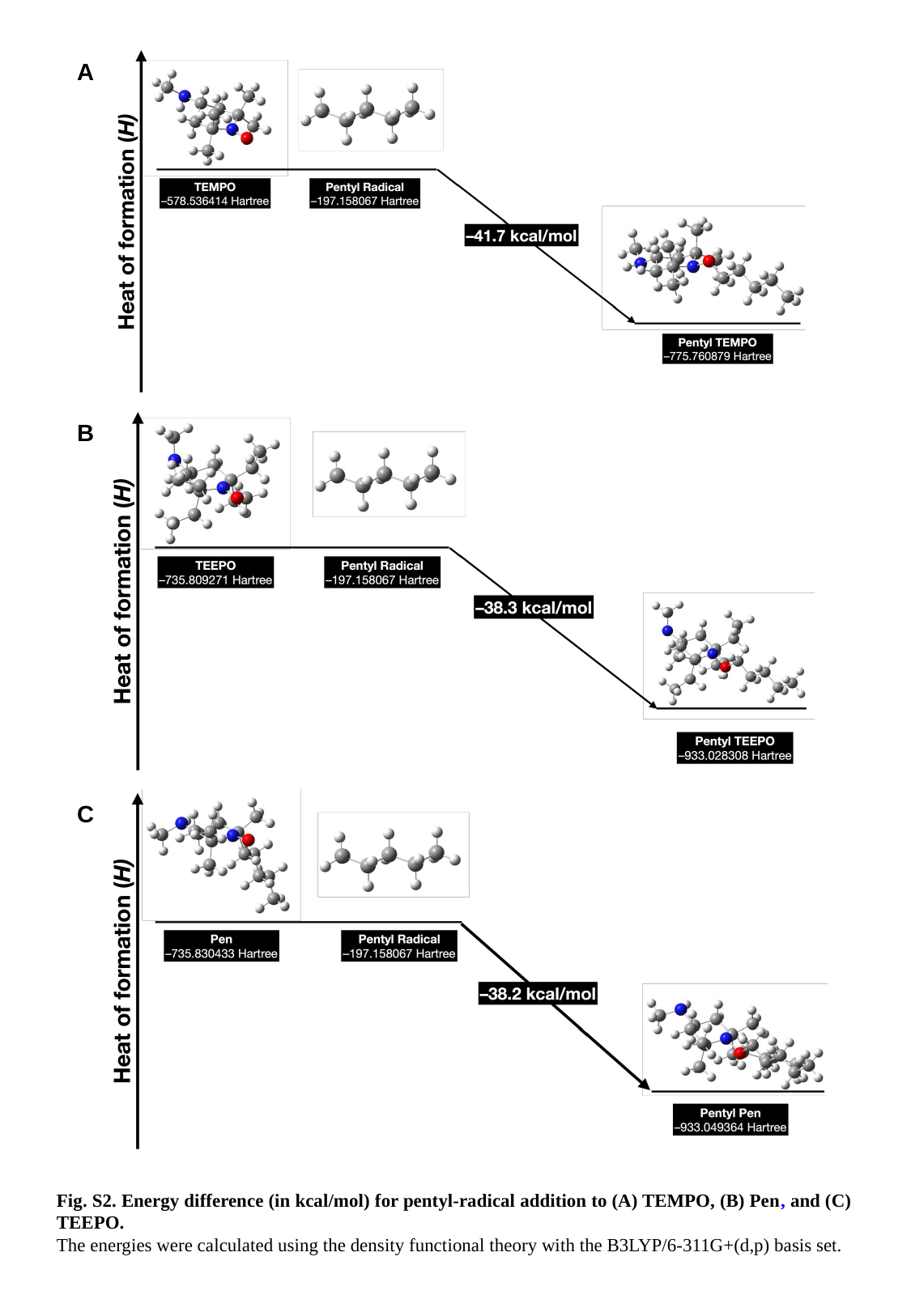

A
B
C
Fig. S2. Energy difference (in kcal/mol) for pentyl-radical addition to (A) TEMPO, (B) Pen, and (C) TEEPO.
The energies were calculated using the density functional theory with the B3LYP/6-311G+(d,p) basis set.

## Slide 3
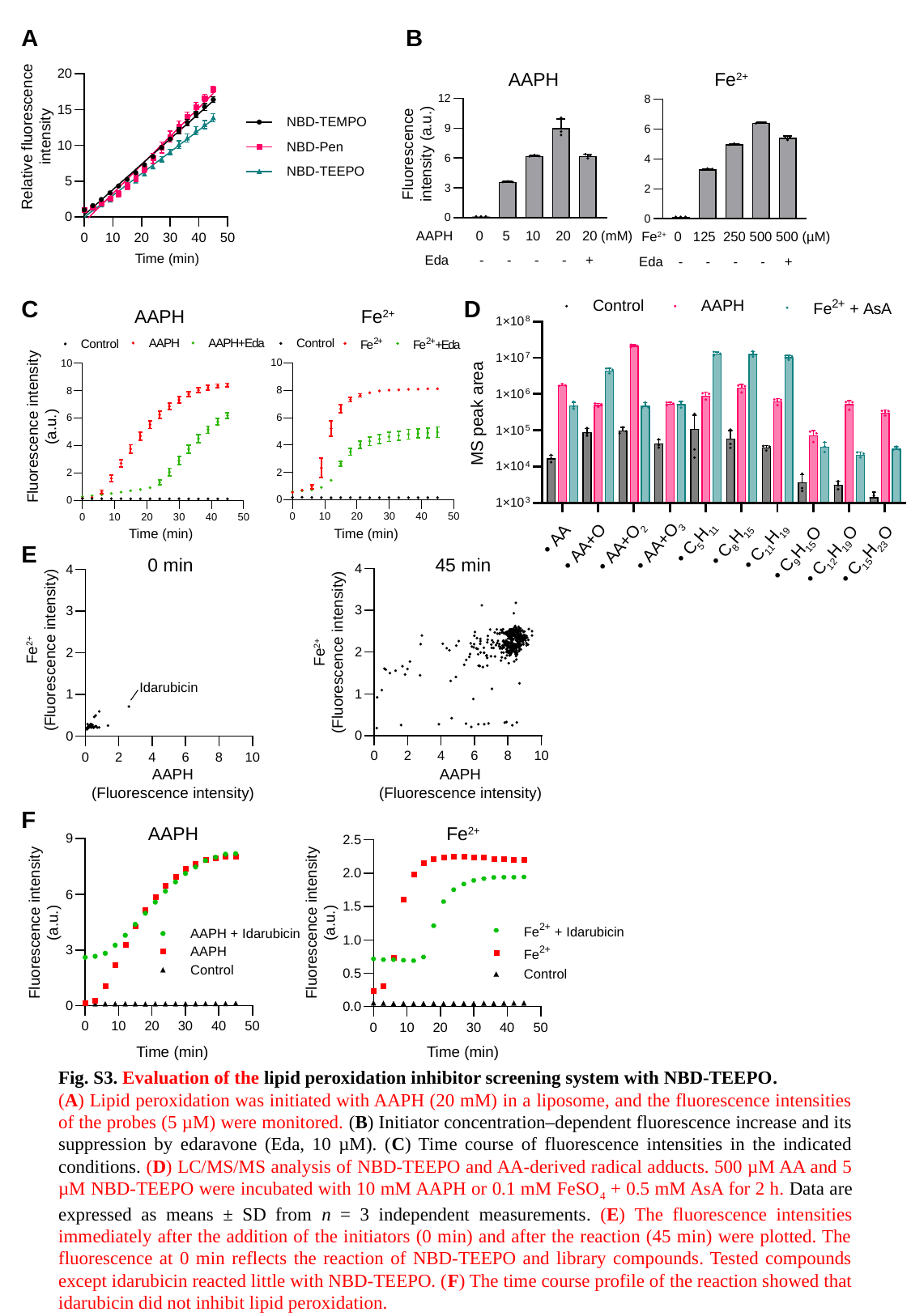

A
B
AAPH
Fe2+
Relative fluorescence intensity
Fluorescence intensity (a.u.)
AAPH 0 5 10 20 20 (mM)
Fe2+ 0 125 250 500 500 (µM)
Time (min)
Eda - - - - +
Eda - - - - +
C
D
Fe2+
AAPH
MS peak area
Fluorescence intensity (a.u.)
Time (min)
Time (min)
 • AA
• C5H11
 • C8H15
 • AA+O3
 • C11H19
 • AA+O2
 • AA+O
E
 • C9H15O
 • C12H19O
 • C15H23O
0 min
45 min
Fe2+
(Fluorescence intensity)
Fe2+
(Fluorescence intensity)
Idarubicin
AAPH
(Fluorescence intensity)
AAPH
(Fluorescence intensity)
F
AAPH
Fe2+
Fluorescence intensity (a.u.)
Fluorescence intensity (a.u.)
Time (min)
Time (min)
Fig. S3. Evaluation of the lipid peroxidation inhibitor screening system with NBD-TEEPO.
(A) Lipid peroxidation was initiated with AAPH (20 mM) in a liposome, and the fluorescence intensities of the probes (5 µM) were monitored. (B) Initiator concentration–dependent fluorescence increase and its suppression by edaravone (Eda, 10 µM). (C) Time course of fluorescence intensities in the indicated conditions. (D) LC/MS/MS analysis of NBD-TEEPO and AA-derived radical adducts. 500 µM AA and 5 µM NBD-TEEPO were incubated with 10 mM AAPH or 0.1 mM FeSO4 + 0.5 mM AsA for 2 h. Data are expressed as means ± SD from n = 3 independent measurements. (E) The fluorescence intensities immediately after the addition of the initiators (0 min) and after the reaction (45 min) were plotted. The fluorescence at 0 min reflects the reaction of NBD-TEEPO and library compounds. Tested compounds except idarubicin reacted little with NBD-TEEPO. (F) The time course profile of the reaction showed that idarubicin did not inhibit lipid peroxidation.

## Slide 4
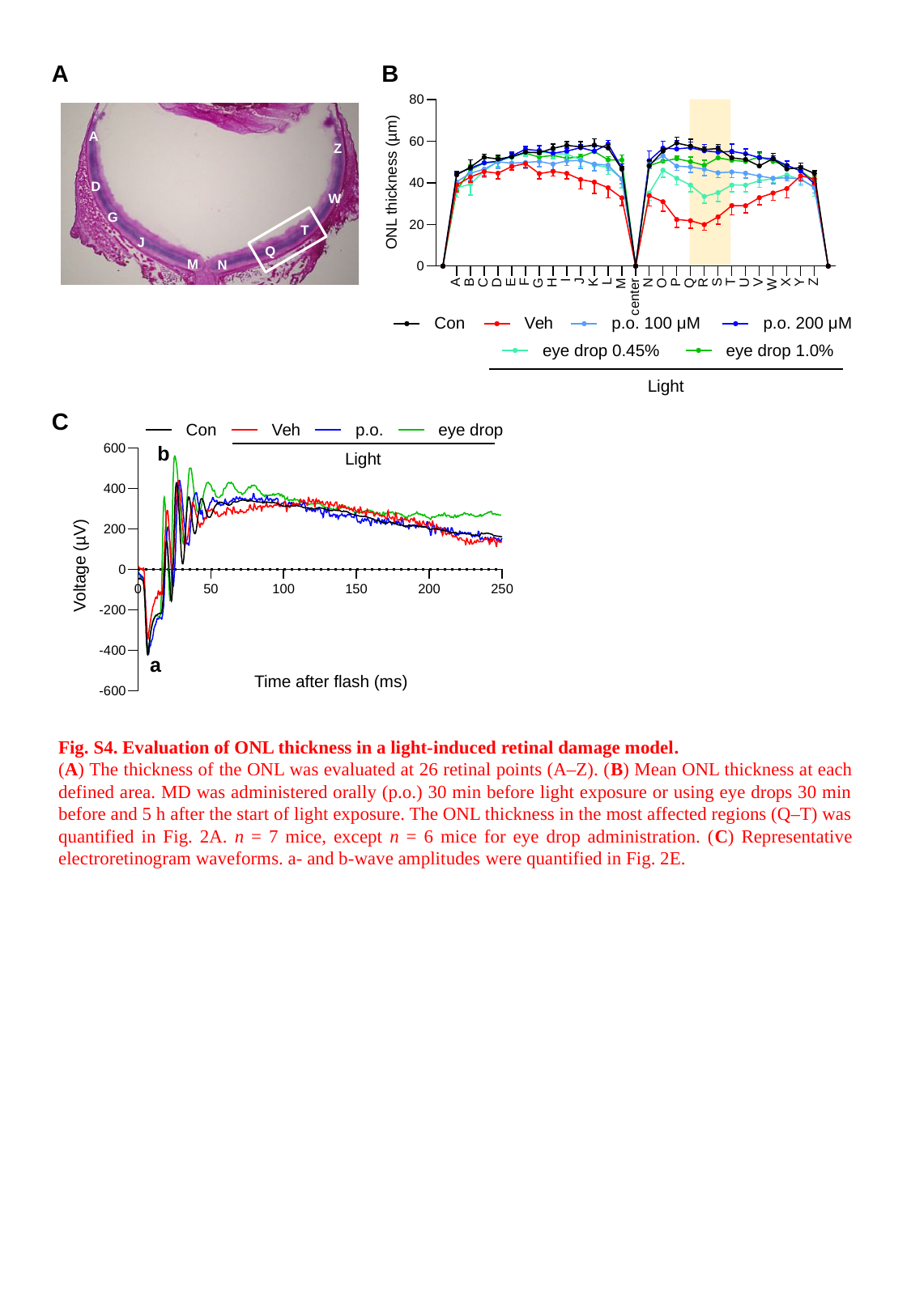

A
B
A
Z
ONL thickness (µm)
D
W
G
T
J
Q
M
N
Light
C
b
Light
Voltage (µV)
a
Time after flash (ms)
Fig. S4. Evaluation of ONL thickness in a light-induced retinal damage model.
(A) The thickness of the ONL was evaluated at 26 retinal points (A–Z). (B) Mean ONL thickness at each defined area. MD was administered orally (p.o.) 30 min before light exposure or using eye drops 30 min before and 5 h after the start of light exposure. The ONL thickness in the most affected regions (Q–T) was quantified in Fig. 2A. n = 7 mice, except n = 6 mice for eye drop administration. (C) Representative electroretinogram waveforms. a- and b-wave amplitudes were quantified in Fig. 2E.

## Slide 5
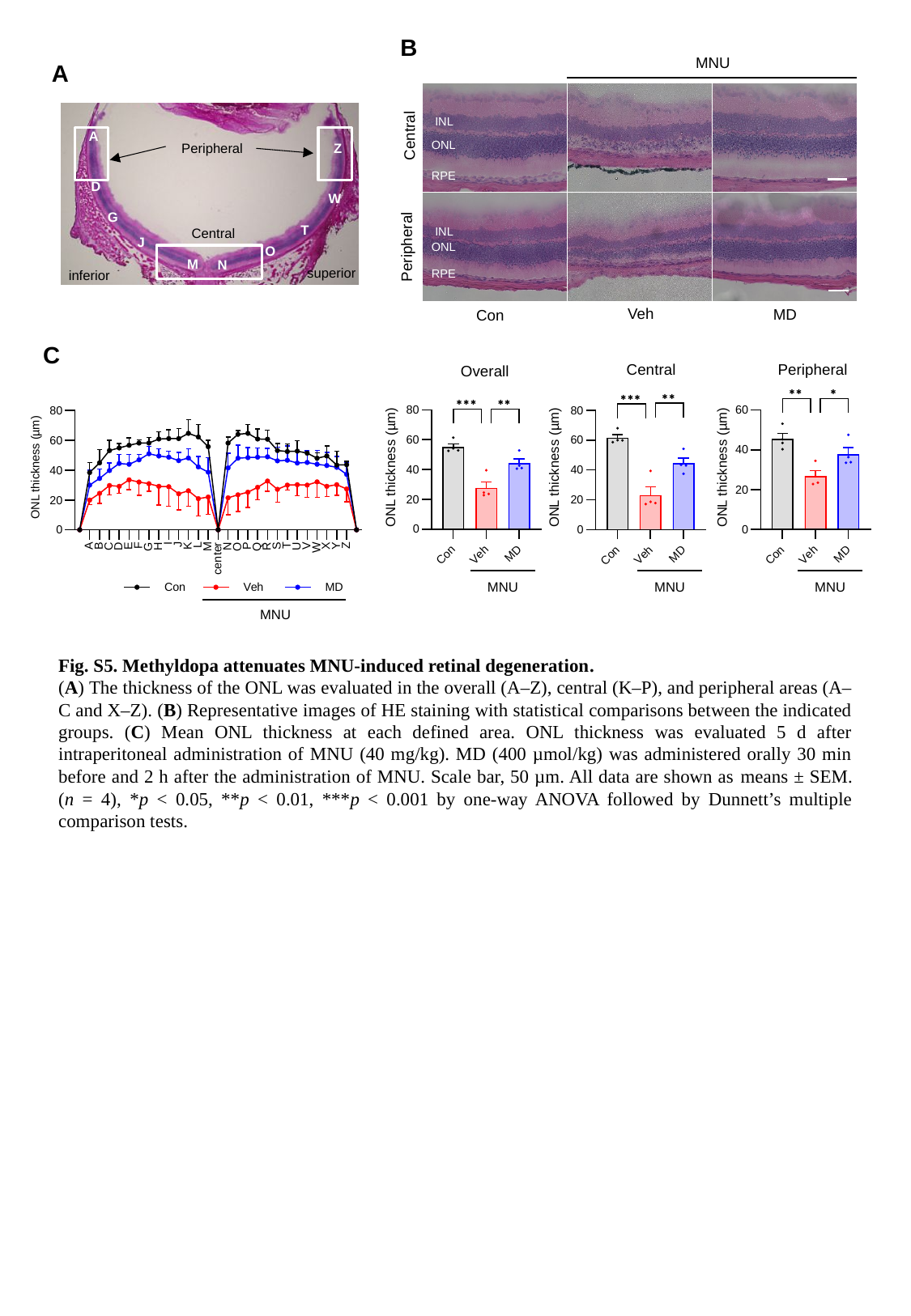

B
MNU
A
INL
Central
A
ONL
Peripheral
Z
RPE
D
W
G
T
INL
Central
J
Peripheral
ONL
O
M
N
superior
RPE
inferior
Veh
MD
Con
C
Peripheral
Central
Overall
ONL thickness (µm)
ONL thickness (µm)
ONL thickness (µm)
ONL thickness (µm)
MNU
MNU
MNU
MNU
Fig. S5. Methyldopa attenuates MNU-induced retinal degeneration.
(A) The thickness of the ONL was evaluated in the overall (A–Z), central (K–P), and peripheral areas (A–C and X–Z). (B) Representative images of HE staining with statistical comparisons between the indicated groups. (C) Mean ONL thickness at each defined area. ONL thickness was evaluated 5 d after intraperitoneal administration of MNU (40 mg/kg). MD (400 µmol/kg) was administered orally 30 min before and 2 h after the administration of MNU. Scale bar, 50 µm. All data are shown as means ± SEM. (n = 4), *p < 0.05, **p < 0.01, ***p < 0.001 by one-way ANOVA followed by Dunnett’s multiple comparison tests.

## Slide 6
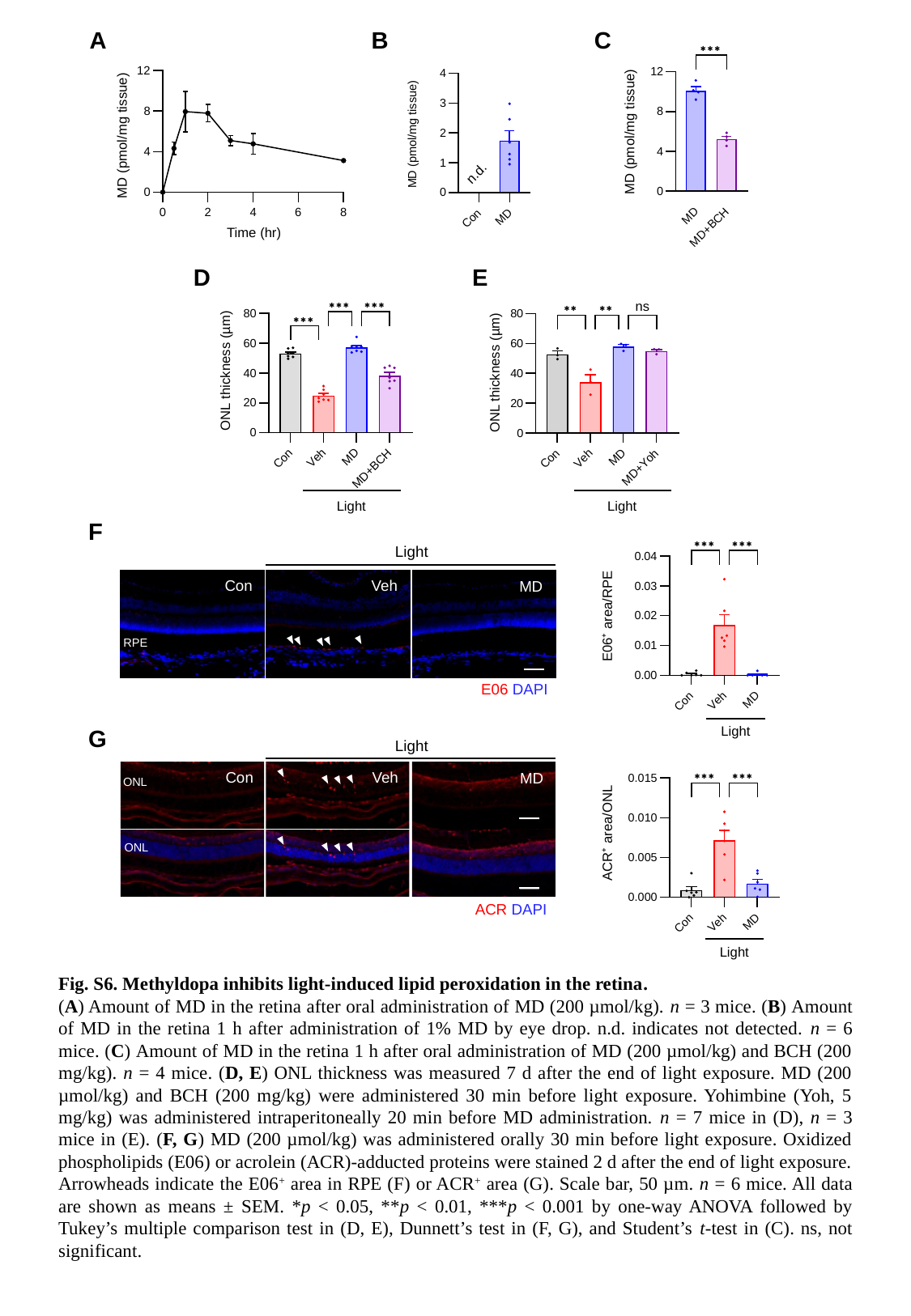

A
B
C
MD (pmol/mg tissue)
MD (pmol/mg tissue)
MD (pmol/mg tissue)
n.d.
Time (hr)
D
E
ONL thickness (µm)
ONL thickness (µm)
Light
Light
F
Light
Con
Veh
MD
E06⁺ area/RPE
RPE
E06 DAPI
Light
G
Light
Con
Veh
MD
ONL
ONL
ONL
ACR⁺ area/ONL
ONL
ACR DAPI
Light
Fig. S6. Methyldopa inhibits light-induced lipid peroxidation in the retina.
(A) Amount of MD in the retina after oral administration of MD (200 µmol/kg). n = 3 mice. (B) Amount of MD in the retina 1 h after administration of 1% MD by eye drop. n.d. indicates not detected. n = 6 mice. (C) Amount of MD in the retina 1 h after oral administration of MD (200 µmol/kg) and BCH (200 mg/kg). n = 4 mice. (D, E) ONL thickness was measured 7 d after the end of light exposure. MD (200 µmol/kg) and BCH (200 mg/kg) were administered 30 min before light exposure. Yohimbine (Yoh, 5 mg/kg) was administered intraperitoneally 20 min before MD administration. n = 7 mice in (D), n = 3 mice in (E). (F, G) MD (200 µmol/kg) was administered orally 30 min before light exposure. Oxidized phospholipids (E06) or acrolein (ACR)-adducted proteins were stained 2 d after the end of light exposure. Arrowheads indicate the E06+ area in RPE (F) or ACR+ area (G). Scale bar, 50 µm. n = 6 mice. All data are shown as means ± SEM. *p < 0.05, **p < 0.01, ***p < 0.001 by one-way ANOVA followed by Tukey’s multiple comparison test in (D, E), Dunnett’s test in (F, G), and Student’s t-test in (C). ns, not significant.

## Slide 7
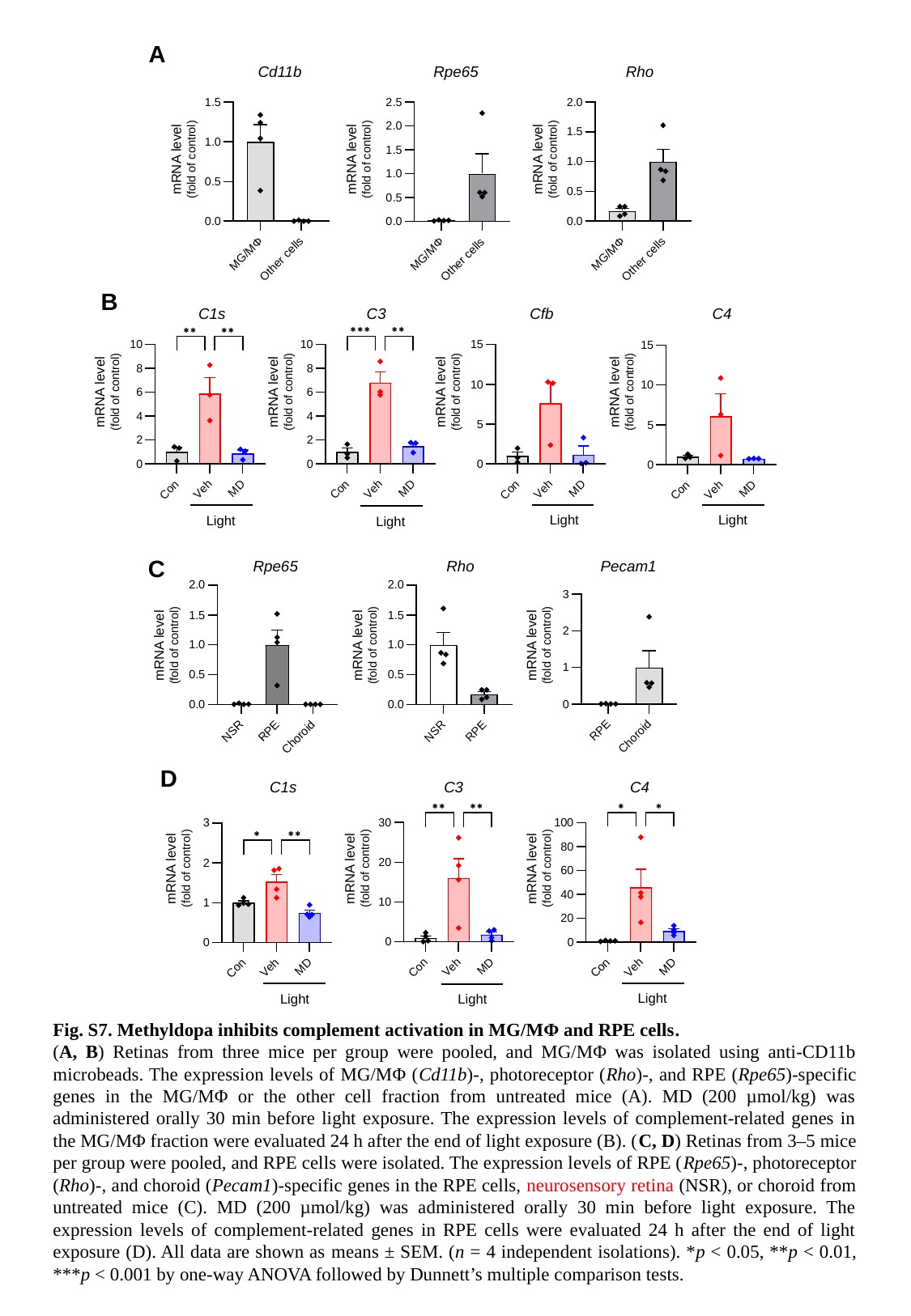

A
Rho
Rpe65
Cd11b
mRNA level
(fold of control)
mRNA level
(fold of control)
mRNA level
(fold of control)
B
C1s
C3
Cfb
C4
mRNA level
(fold of control)
mRNA level
(fold of control)
mRNA level
(fold of control)
mRNA level
(fold of control)
Light
Light
Light
Light
C
Rpe65
Rho
Pecam1
mRNA level
(fold of control)
mRNA level
(fold of control)
mRNA level
(fold of control)
D
C1s
C3
C4
mRNA level
(fold of control)
mRNA level
(fold of control)
mRNA level
(fold of control)
Light
Light
Light
Fig. S7. Methyldopa inhibits complement activation in MG/MΦ and RPE cells.
(A, B) Retinas from three mice per group were pooled, and MG/MΦ was isolated using anti-CD11b microbeads. The expression levels of MG/MΦ (Cd11b)-, photoreceptor (Rho)-, and RPE (Rpe65)-specific genes in the MG/MΦ or the other cell fraction from untreated mice (A). MD (200 µmol/kg) was administered orally 30 min before light exposure. The expression levels of complement-related genes in the MG/MΦ fraction were evaluated 24 h after the end of light exposure (B). (C, D) Retinas from 3–5 mice per group were pooled, and RPE cells were isolated. The expression levels of RPE (Rpe65)-, photoreceptor (Rho)-, and choroid (Pecam1)-specific genes in the RPE cells, neurosensory retina (NSR), or choroid from untreated mice (C). MD (200 µmol/kg) was administered orally 30 min before light exposure. The expression levels of complement-related genes in RPE cells were evaluated 24 h after the end of light exposure (D). All data are shown as means ± SEM. (n = 4 independent isolations). *p < 0.05, **p < 0.01, ***p < 0.001 by one-way ANOVA followed by Dunnett’s multiple comparison tests.

## Slide 8
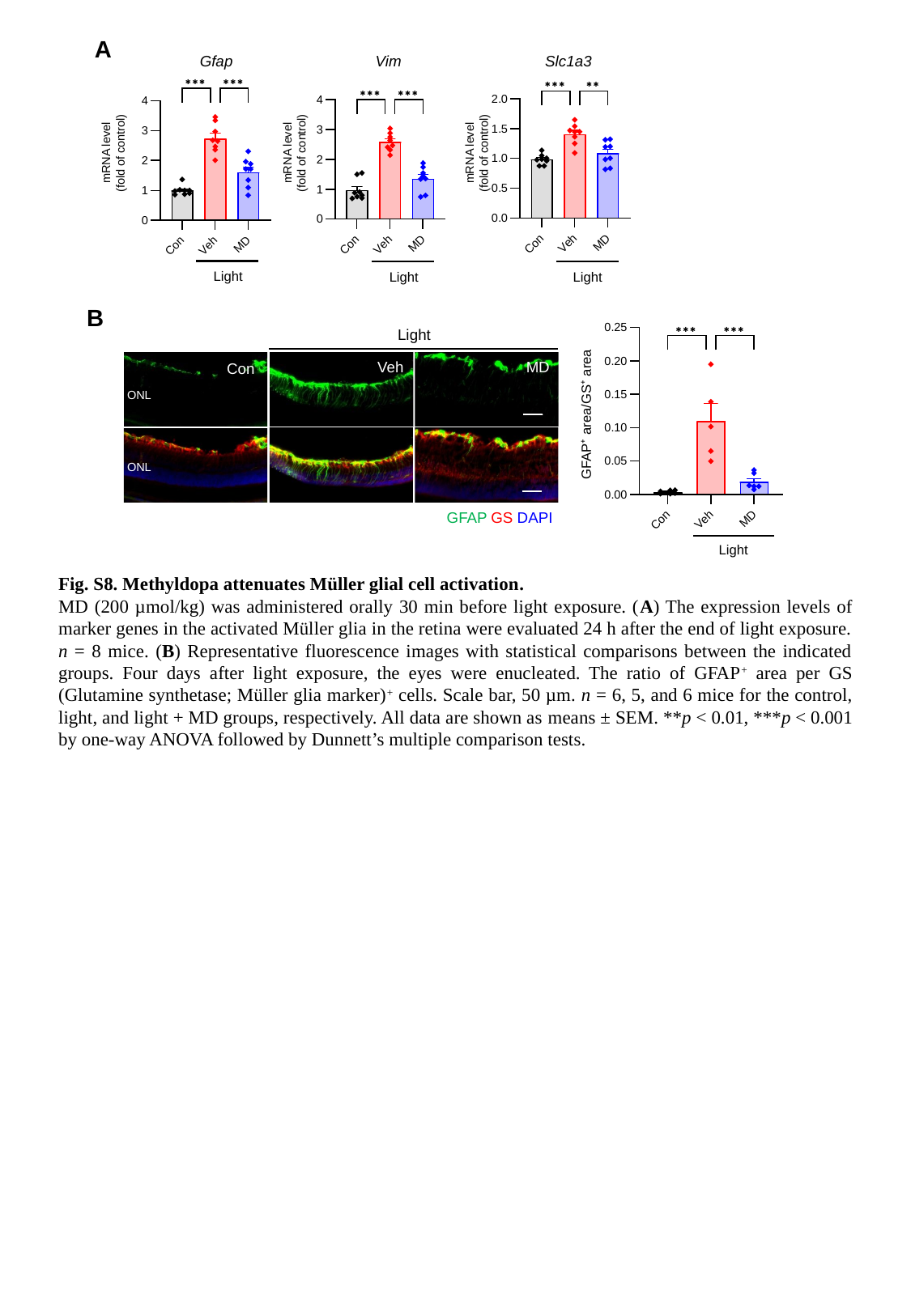

A
Gfap
Vim
Slc1a3
mRNA level
(fold of control)
mRNA level
(fold of control)
mRNA level
(fold of control)
Light
Light
Light
B
Light
Veh
MD
Con
ONL
GFAP⁺ area/GS⁺ area
ONL
GFAP GS DAPI
Light
Fig. S8. Methyldopa attenuates Müller glial cell activation.
MD (200 µmol/kg) was administered orally 30 min before light exposure. (A) The expression levels of marker genes in the activated Müller glia in the retina were evaluated 24 h after the end of light exposure. n = 8 mice. (B) Representative fluorescence images with statistical comparisons between the indicated groups. Four days after light exposure, the eyes were enucleated. The ratio of GFAP+ area per GS (Glutamine synthetase; Müller glia marker)+ cells. Scale bar, 50 µm. n = 6, 5, and 6 mice for the control, light, and light + MD groups, respectively. All data are shown as means ± SEM. **p < 0.01, ***p < 0.001 by one-way ANOVA followed by Dunnett’s multiple comparison tests.

## Slide 9
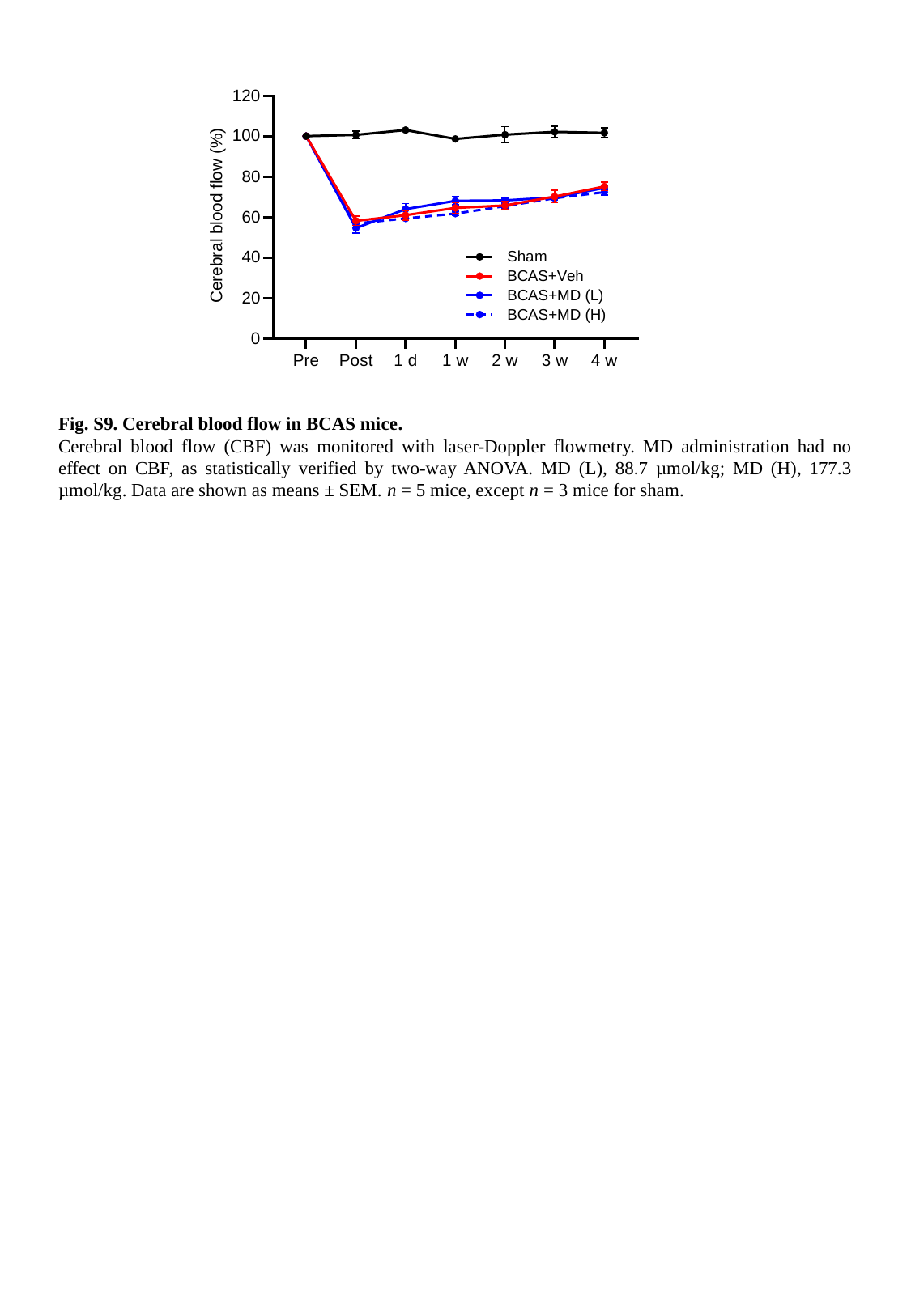

Cerebral blood flow (%)
Fig. S9. Cerebral blood flow in BCAS mice.
Cerebral blood flow (CBF) was monitored with laser-Doppler flowmetry. MD administration had no effect on CBF, as statistically verified by two-way ANOVA. MD (L), 88.7 µmol/kg; MD (H), 177.3 µmol/kg. Data are shown as means ± SEM. n = 5 mice, except n = 3 mice for sham.

## Slide 10
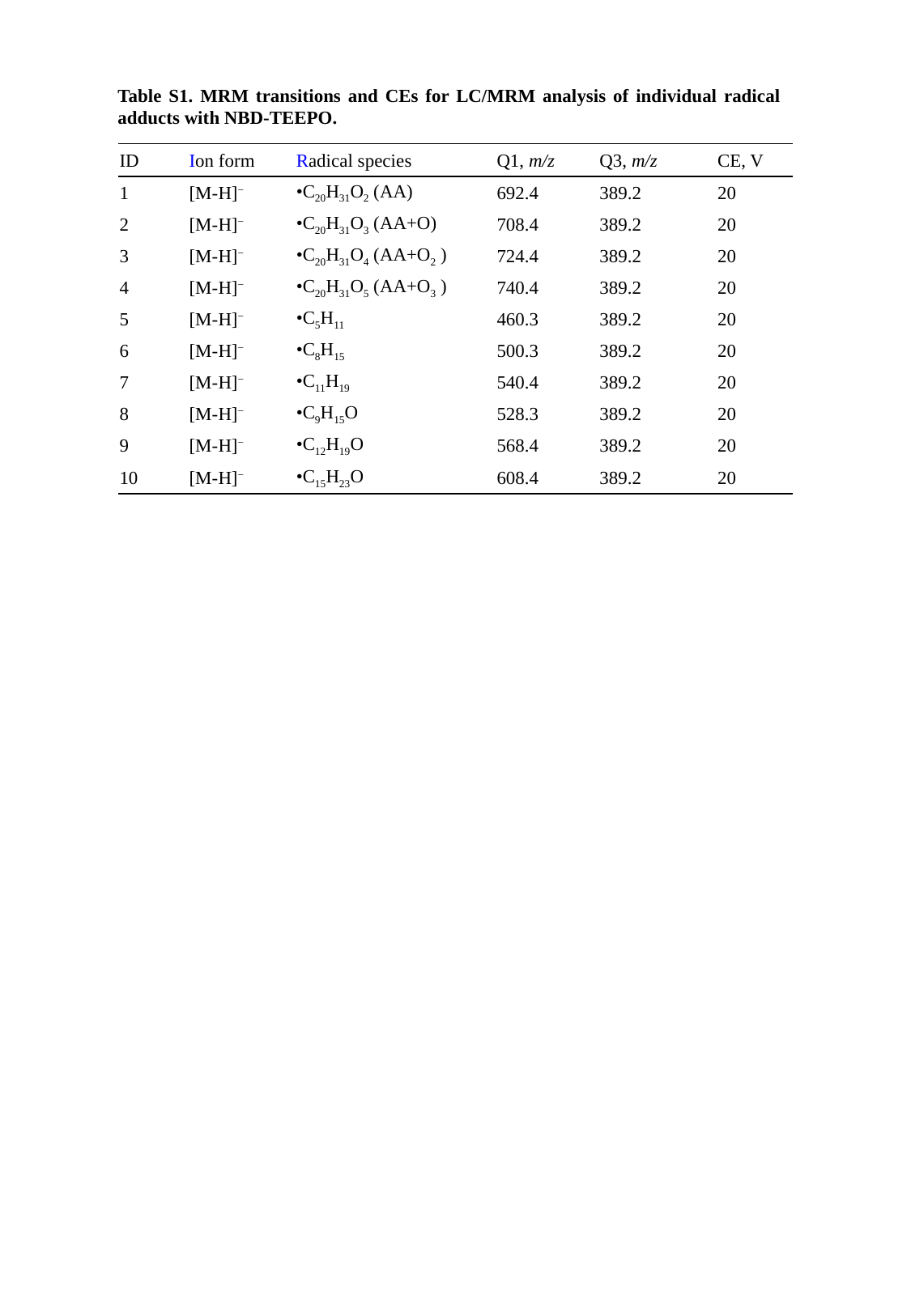

Table S1. MRM transitions and CEs for LC/MRM analysis of individual radical adducts with NBD-TEEPO.
| ID | Ion form | Radical species | Q1, m/z | Q3, m/z | CE, V |
| --- | --- | --- | --- | --- | --- |
| 1 | [M-H]− | •C20H31O2 (AA) | 692.4 | 389.2 | 20 |
| 2 | [M-H]− | •C20H31O3 (AA+O) | 708.4 | 389.2 | 20 |
| 3 | [M-H]− | •C20H31O4 (AA+O2 ) | 724.4 | 389.2 | 20 |
| 4 | [M-H]− | •C20H31O5 (AA+O3 ) | 740.4 | 389.2 | 20 |
| 5 | [M-H]− | •C5H11 | 460.3 | 389.2 | 20 |
| 6 | [M-H]− | •C8H15 | 500.3 | 389.2 | 20 |
| 7 | [M-H]− | •C11H19 | 540.4 | 389.2 | 20 |
| 8 | [M-H]− | •C9H15O | 528.3 | 389.2 | 20 |
| 9 | [M-H]− | •C12H19O | 568.4 | 389.2 | 20 |
| 10 | [M-H]− | •C15H23O | 608.4 | 389.2 | 20 |

## Slide 11
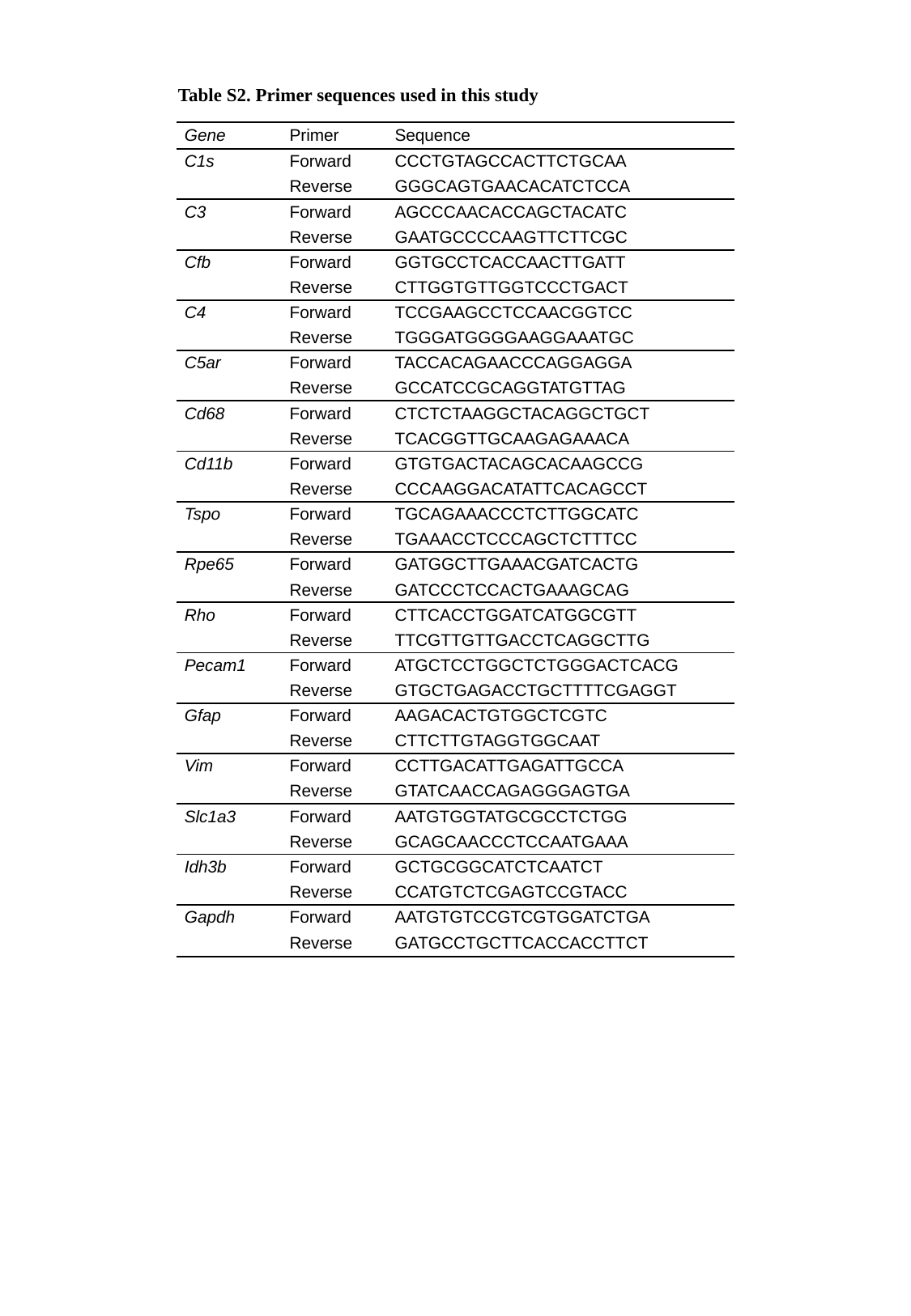

Table S2. Primer sequences used in this study
| Gene | Primer | Sequence |
| --- | --- | --- |
| C1s | Forward | CCCTGTAGCCACTTCTGCAA |
| | Reverse | GGGCAGTGAACACATCTCCA |
| C3 | Forward | AGCCCAACACCAGCTACATC |
| | Reverse | GAATGCCCCAAGTTCTTCGC |
| Cfb | Forward | GGTGCCTCACCAACTTGATT |
| | Reverse | CTTGGTGTTGGTCCCTGACT |
| C4 | Forward | TCCGAAGCCTCCAACGGTCC |
| | Reverse | TGGGATGGGGAAGGAAATGC |
| C5ar | Forward | TACCACAGAACCCAGGAGGA |
| | Reverse | GCCATCCGCAGGTATGTTAG |
| Cd68 | Forward | CTCTCTAAGGCTACAGGCTGCT |
| | Reverse | TCACGGTTGCAAGAGAAACA |
| Cd11b | Forward | GTGTGACTACAGCACAAGCCG |
| | Reverse | CCCAAGGACATATTCACAGCCT |
| Tspo | Forward | TGCAGAAACCCTCTTGGCATC |
| | Reverse | TGAAACCTCCCAGCTCTTTCC |
| Rpe65 | Forward | GATGGCTTGAAACGATCACTG |
| | Reverse | GATCCCTCCACTGAAAGCAG |
| Rho | Forward | CTTCACCTGGATCATGGCGTT |
| | Reverse | TTCGTTGTTGACCTCAGGCTTG |
| Pecam1 | Forward | ATGCTCCTGGCTCTGGGACTCACG |
| | Reverse | GTGCTGAGACCTGCTTTTCGAGGT |
| Gfap | Forward | AAGACACTGTGGCTCGTC |
| | Reverse | CTTCTTGTAGGTGGCAAT |
| Vim | Forward | CCTTGACATTGAGATTGCCA |
| | Reverse | GTATCAACCAGAGGGAGTGA |
| Slc1a3 | Forward | AATGTGGTATGCGCCTCTGG |
| | Reverse | GCAGCAACCCTCCAATGAAA |
| Idh3b | Forward | GCTGCGGCATCTCAATCT |
| | Reverse | CCATGTCTCGAGTCCGTACC |
| Gapdh | Forward | AATGTGTCCGTCGTGGATCTGA |
| | Reverse | GATGCCTGCTTCACCACCTTCT |

## Slide 12
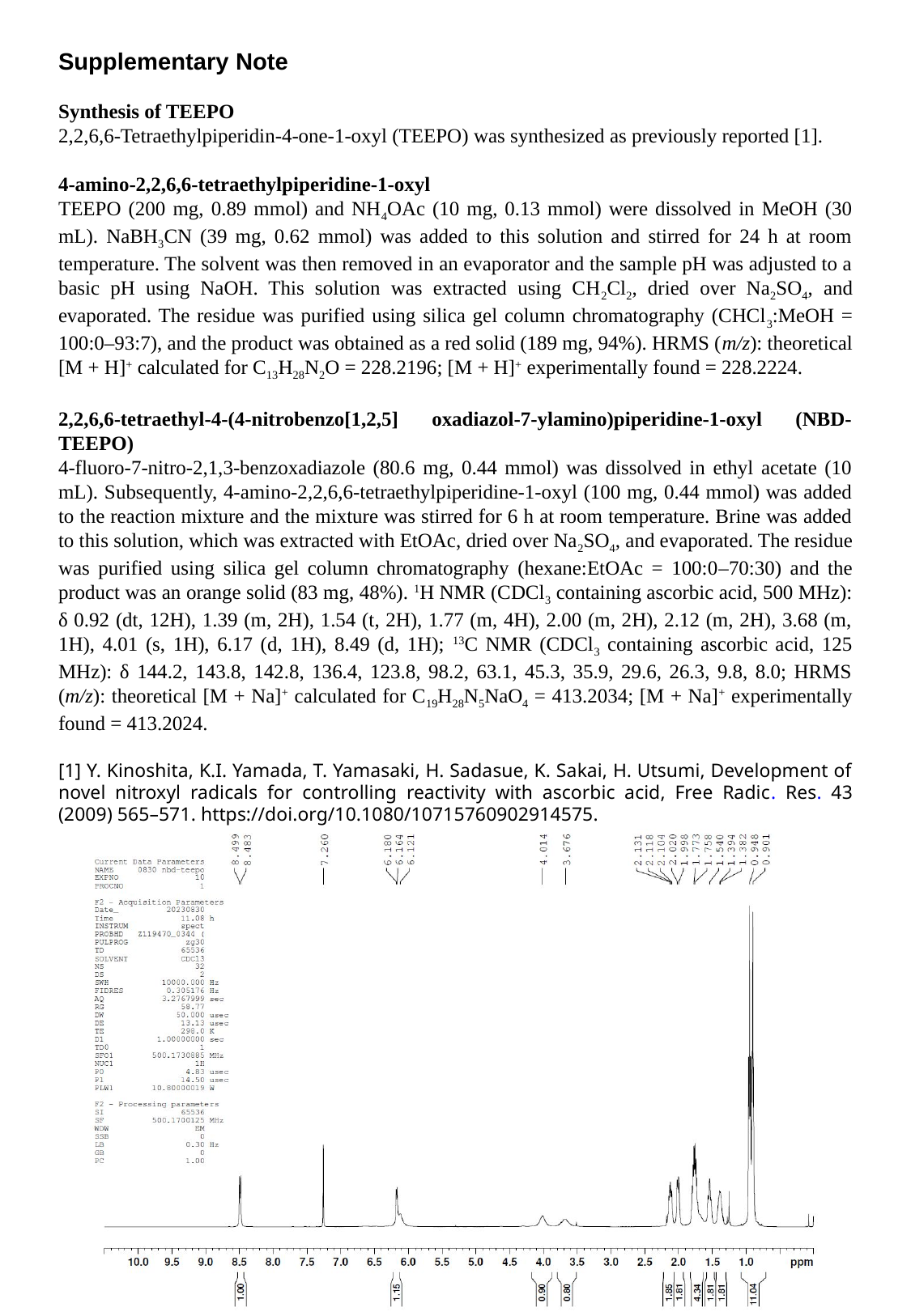

Supplementary Note
Synthesis of TEEPO
2,2,6,6-Tetraethylpiperidin-4-one-1-oxyl (TEEPO) was synthesized as previously reported [1].
4-amino-2,2,6,6-tetraethylpiperidine-1-oxyl
TEEPO (200 mg, 0.89 mmol) and NH4OAc (10 mg, 0.13 mmol) were dissolved in MeOH (30 mL). NaBH3CN (39 mg, 0.62 mmol) was added to this solution and stirred for 24 h at room temperature. The solvent was then removed in an evaporator and the sample pH was adjusted to a basic pH using NaOH. This solution was extracted using CH2Cl2, dried over Na2SO4, and evaporated. The residue was purified using silica gel column chromatography (CHCl3:MeOH = 100:0–93:7), and the product was obtained as a red solid (189 mg, 94%). HRMS (m/z): theoretical [M + H]+ calculated for C13H28N2O = 228.2196; [M + H]+ experimentally found = 228.2224.
2,2,6,6-tetraethyl-4-(4-nitrobenzo[1,2,5] oxadiazol-7-ylamino)piperidine-1-oxyl (NBD-TEEPO)
4-fluoro-7-nitro-2,1,3-benzoxadiazole (80.6 mg, 0.44 mmol) was dissolved in ethyl acetate (10 mL). Subsequently, 4-amino-2,2,6,6-tetraethylpiperidine-1-oxyl (100 mg, 0.44 mmol) was added to the reaction mixture and the mixture was stirred for 6 h at room temperature. Brine was added to this solution, which was extracted with EtOAc, dried over Na2SO4, and evaporated. The residue was purified using silica gel column chromatography (hexane:EtOAc = 100:0–70:30) and the product was an orange solid (83 mg, 48%). 1H NMR (CDCl3 containing ascorbic acid, 500 MHz): δ 0.92 (dt, 12H), 1.39 (m, 2H), 1.54 (t, 2H), 1.77 (m, 4H), 2.00 (m, 2H), 2.12 (m, 2H), 3.68 (m, 1H), 4.01 (s, 1H), 6.17 (d, 1H), 8.49 (d, 1H); 13C NMR (CDCl3 containing ascorbic acid, 125 MHz): δ 144.2, 143.8, 142.8, 136.4, 123.8, 98.2, 63.1, 45.3, 35.9, 29.6, 26.3, 9.8, 8.0; HRMS (m/z): theoretical [M + Na]+ calculated for C19H28N5NaO4 = 413.2034; [M + Na]+ experimentally found = 413.2024.
[1] Y. Kinoshita, K.I. Yamada, T. Yamasaki, H. Sadasue, K. Sakai, H. Utsumi, Development of novel nitroxyl radicals for controlling reactivity with ascorbic acid, Free Radic. Res. 43 (2009) 565–571. https://doi.org/10.1080/10715760902914575.
