# Supplementary material for: Construction of a screening system for lipid-derived radical inhibitors and validation of hit compounds to target retinal and cerebrovascular diseases
Source: Redox Biol. 2024 May 8;73:103186. doi: 10.1016/j.redox.2024.103186 (PMC11109892; doi:10.1016/j.redox.2024.103186)
Supplement: Multimedia component 2 [file mmc2.docx]

**Responses to the Comments from Reviewer #1**

We thank you for your valuable review and helpful suggestions for improving our manuscript. Your comments are listed below in blue, followed by our point-by-point responses. The revised text in the manuscript is shown in red.

(1). The probe tested herein has been reported in patents but this is one of the first manuscripts describing the synthesis and testing the hypothesis that this probe detects lipid derived radicals. As such, the authors need to present confirmation of synthesis (NMR data, etc). Further, the authors need to show proof of concept that the probe is actually responding to lipid oxidation products. Side by side analyses should have been conducted in which the authors measured some type of actual lipid product (HODEs, HETEs, F2-Isoprostanes). This would make the figure showing the increase of fluorescence withe AAPH concentration (Supplement Figure 1) more convincing. Further, side by side comparisons should have been made in the animal model. If the authors truly want to use this as a screening probe for lipid peroxidation products, the proof of concept needs to be demonstrated.

For NBD-Pen and NBD-TEMPO, the synthesis methods and properties have been reported in a previous publication (Ref. 10; Yamada *et al., Nat Chem Biol,* 2016). However, as pointed out, NBD-TEEPO is a novel probe. In the previous version of the current manuscript, we presented the NMR data for NBD-TEEPO in the Materials and Methods section. To make this information more accessible, we have now moved it to the Supplementary Material and have also added the NMR spectra for further clarity.

In this study, we developed NBD-TEEPO to efficiently search for radical-trapping antioxidants (RTAs) *in vitro*. In contrast, we previously demonstrated that NBD-Pen can efficiently detect lipid radicals *in vivo* (Ref. 10; Yamada *et al., Nat Chem Biol,* 2016). In addition, we successfully used NBD-Pen to detect lipid radicals in the retina (**Fig. 2H**).

NBD-TEEPO is a probe that reacts specifically with lipid radicals and does not react with lipid products such as HODEs, HETEs, and F2-Isoprostanes. In the RTA screening experiment (**Fig. 1**), NBD-TEEPO detected lipid radicals derived from phosphatidylcholine, which constitutes liposomes. To more directly demonstrate that NBD-TEEPO detects lipid radicals, we showed that NBD-TEEPO forms adducts with arachidonic acid (AA)-derived radicals. In the figure below (added as **Fig. S3D**), AA-derived lipid radicals were generated by AAPH (a radical initiator) or Fe^2+^ ion, and NBD-TEEPO–radical adducts were quantified via LC/MS/MS. This result indicates that multiple species of lipid radicals, including oxygenated and fragmented radicals, were captured by NBD-TEEPO.

**Fig. S3.** (**D**) LC/MS/MS analysis of NBD-TEEPO and AA-derived radical adducts. 500 µM AA and 5 µM NBD-TEEPO were incubated with 10 mM AAPH or 0.1 mM FeSO_4_ + 0.5 mM AsA for 2 h. Data are expressed as means ± SD from *n* = 3 independent measurements.

(2) Most of the figures in the manuscript refer to experiments that don't actually measure any sort of lipid oxidation or lipid radical. The in vivo experiments measure complement activation and other factors that can be associated with lipid oxidation and/or oxidative stress but are not direct measures of what this probe or the methyldopa drug is doing. Until the mechanism is confirmed, this manuscript only implies affecting of lipid radicals.

We apologize for any lack of clarity in our figures. Using NBD-Pen, we demonstrated that lipid radicals were generated in the light-irradiated retina and that their formation was suppressed by methyldopa (**Fig. 2H**). We also showed that the production of oxidized phosphatidylcholine and acrolein, an end-product of lipid peroxidation, was inhibited by methyldopa (**Fig. S6F and G**).

**Fig. 2.** (**H**) NBD-Pen (5.0 µmol/kg) was administered intraperitoneally at the end of light exposure. After 30 min, the eyes were enucleated. *n* = 8 mice. Scale bar, 50 µm.

**Fig. S6** (F, G) MD (200 µmol/kg) was administered orally 30 min before light exposure. Oxidized phospholipids (E06) or acrolein (ACR)-adducted proteins were stained 2 d after the end of light exposure. Arrowheads indicate the E06^+^ area in RPE (F) or ACR^+^ area (G). Scale bar, 50 µm. *n* = 6 mice. All data are shown as means ± SEM. ****p* < 0.001 by one-way ANOVA followed by Dunnett’s tests.

(3) Manuscript needs to be reviewed for grammatical errors.

We apologize for any inaccuracies in grammar. The manuscript has been thoroughly rechecked and revised to improve language quality.

**Responses to the Comments from Reviewer #2**

We thank you for your valuable review and helpful suggestions for improving our manuscript. Your comments are listed below in blue, followed by our point-by-point responses. The revised text in the manuscript is shown in red.

- Line 112 The a-substitution adjacent to the nitroxide moiety is key to the screening methodology and perhaps a sentence could be added explaining the chemistry (i.e. tetramethyl vs. tetraethyl vs trimethylalkyl substituted and ring class of nitroxide) and citing the relevant literature on how this impacts reduction rates by antioxidants and in vivo. Some of this is no doubt discussed in References 10 and 46 but it would be good to outline this to the Redox Biology audience.

We agree with this point. We have added the following text to explain the effect of α-substitution on the reactivity of the probes.

[Line 123] We have previously reported that substitution at the α-position of piperidine nitroxides affects their reactivity with reducing antioxidants by altering the steric and electronic environments around the N-O moiety [14] and that the inhibitory activity of nitroxides against lipid peroxidation depends on their lipophilicity [15]. In this study, we synthesized the fluorescent probe 2,2,6,6-tetraethyl-4-(4-nitrobenzo[1,2,5]oxadiazol-7-ylamino)piperidine-1-oxyl (NBD-TEEPO), which exhibits minimal reactivity with antioxidants and displays high specificity for lipid radicals.

- Line 117/385 Why was the tetra-ethyl probe chosen? Nitroxides reduce in vivo, however the authors and others have shown that this can be hindered/slowed with the addition of steric bulk around the nitroxide moiety. It is also impacted by the localisation, type of tissue and/or metabolic state. An important control measurement therefore is to know the rate of fluorescence generation (if any) in the absence of initiator and/or test compounds and whether this is affected by the environment. Were such controls undertaken? If so, this could be an important contribution to the study even if the details are only present in the SI. At the moment SI Fig1 Panel A seems to relate to this. Were these experiments undertaken in liposomes? Does the lipophilicity of the probe have an impact on the response? Are the three control experiments for the three PFNs of similar fluorescence response and does this change with time?

We have reported that there is a correlation between the lipophilicity of the probe and its reactivity with lipid radicals (Ref. 15; Yamasaki *et al. J Org Chem* 2011) and that NBD-Pen, which is highly lipophilic, selectively reacts with lipid radicals (Ref. 10; Yamada *et al., Nat Chem Biol,* 2016).

While the clogP of NBD-TEMPO is 1.7, both NBD-Pen and NBD-TEEPO have a clogP of 3.8. Three nitroxide probes showed similar reactivity in the liposome system (added as **Fig. S3A**). Furthermore, NBD-TEEPO did not react with reducing antioxidants (**Fig. S1**). Therefore, we selected NBD-TEEPO as the *in vitro* screening probe. The RTA screening experiments, including those shown in **Fig. S1A and B**, were performed in liposomes, reflecting the reaction of the probes in a lipophilic environment.

**Fig. S3B and C** (S1C and D in the previous version of the manuscript) show the fluorescence of the probe without the initiators or test compounds, indicating that the fluorescence did not change in the absence of the initiators and that the fluorescence increased depending on the presence of the initiator.

The time course of the reaction of the three probes with reducing antioxidants has been included in **Fig. S1A**. The probes reacted rapidly with most of the antioxidants, but the reaction between NBD-TEMPO and AsA proceeded slowly thereafter. This reaction is quite different from the reaction with lipid radicals, which takes more than 40 min to reach a plateau.

In this study, we developed NBD-TEEPO to efficiently search for RTAs *in vitro*. We previously demonstrated that NBD-Pen can efficiently detect lipid radicals *in vivo* (Ref. 10; Yamada *et al., Nat Chem Biol,* 2016). In addition, we successfully used NBD-Pen to detect lipid radicals in the retina (**Fig. 2H**).

**Fig. S3** (**A**) Lipid peroxidation was initiated with AAPH (20 mM) in a liposome, and the fluorescence intensities of the two probes (5 µM) were monitored.

**Fig. S3** (**B**) Initiator concentration–dependent fluorescence increase and its suppression by edaravone (Eda, 10 µM). (**C**) Time course of fluorescence intensities in the indicated conditions.

**Fig. S1** (**A**) Time course of fluorescence intensities of 5 µM NBD-TEMPO, NBD-Pen, or NBD-TEEPO mixed with 50 µM ascorbic acid (AsA), uric acid (UA), chlorogenic acid (CGA), edaravone (Eda), ferrostatin-1 (Fer-1), Trolox (Tro), or vitamin E (VitE).

We have revised the following text regarding the lipophilicity and reactivity of NBD-TEEPO as follows:

[Line 411] While the inhibitory activity of nitroxides against lipid peroxidation depends on their lipophilicity, clogP values of NBD-Pen and NBD-TEEPO are comparable and higher than that of NBD-TEMPO [15].

[Line 418] The fluorescence intensity of NBD-TEEPO did not change in the absence of lipid radical initiators and increased in a concentration- and time-dependent manner in the presence of initiators (**Fig. S3B and C**).

- Line 444 Of relevance, and missing from the literature cited, are studies from the Barnett group relating to the use of a not unrelated profluorescent nitroxide technique to assess retinal function. Experimental Eye Research (2014), 129, 48-56, Neurochemical Research (2023), 48(11), 3402-3419, Neurochemistry International (2016), 92, 1-12, Free Radical Biology & Medicine (2018), 128, 97-110. Much of this research seems to be very relevant to the current screening method, particularly with respect to oxidative damage to the retina.

To strengthen the validity of the application of this method to retinal injury models, we have cited the recommended publications as follows:

[Line 468] In addition, increased oxidative stress was monitored in a retinal injury model using a profluorescent nitroxide probe [35–37].

- Line 397 “The reactivities of these fluorescent probes toward carbon-centered radicals were evaluated based on the heat of formation…[and]… calculated using density functional theory….. indicating that all three probes are comparable.” The authors later used fluorescence intensity to experimentally monitor activity/reactivity and it is unfortunate that a comparative study using fluorescence intensity was therefore not undertaken to determine the validity of the calculations given that the compounds were in hand.

As mentioned above, we have compared the reactivity of the three nitroxide probes in the revised manuscript. Our results revealed that the reactivity of the three probes is comparable. We have added the following figure and text.

**Fig. S3** (**A**) Lipid peroxidation was initiated with AAPH (20 mM) in a liposome, and the fluorescence intensities of the probes (5 µM) were monitored.

[Line 397] In fact, NBD-Pen and NBD-TEEPO showed comparable rates of fluorescence increase in the liposome system using 2,2′-azobis(2-methylpropionamidine) dihydrochloride (AAPH) as **a** lipid peroxidation initiator **(**Fig. S3A).

- Line 385 Another important control is to ensure that the probe and library of compounds tested do not react directly with each other and provide false-negative / false-positive results. This seems unlikely looking at the structures of the compounds tested, however the authors also indicate that this is an important aspect in the introduction, yet it does not seem as if this was actually tested for all the drugs screened? My reading of the discussion in the manuscript doesn’t seem to indicate that this was confirmed.

We apologize for any ambiguity regarding false negative or false positive results. We have shown the fluorescence intensities immediately after the addition of initiators in **Fig. S3E**. Almost all compounds exhibited no increase in fluorescence intensity, indicating that they did not directly react with NBD-TEEPO. Even for idarubicin, which showed the largest increase in fluorescence intensity, the increase was negligible compared to the increase in fluorescence after addition of the initiator (**Fig. S3F**). Therefore, it is presumed that there are no false negatives due to the reaction of NBD-TEEPO with library compounds. We have added the following text to clarify this point in the revised manuscript.

[Line 433] All test compounds, except idarubicin, did not directly react with NBD-TEEPO, and the reaction between idarubicin and NBD-TEEPO was negligible, thus eliminating the possibility of a false negative result (**Fig. S3E and F**).

- Line 227. I don’t think that the protocol for MNU-induced retinal degeneration is described in Ref 52. Could the authors please provide an appropriate reference? Also it would be better if MNU is defined here at the first instance, not later (at L471).

We apologize for the oversight regarding the reference cited. Another paper was incorrectly cited, and the citation number was also incorrect. These have been corrected now, and the following report has been cited as Ref. 23. MNU has also been defined at its first mention (in line 234).

[23] S.B. Smith, K.L. Yielding, Retinal degeneration in the mouse. A model induced transplaceatally by methylnitrosourea, Exp. Eye Res. 43 (1986) 791–801. https://doi.org/10.1016/S0014-4835(86)80010-0.

- Line 246 ERG. The unit of luminous intensity for flash stimuli should be expressed cd.s.m^-2^

(candela second per square metre). The ERG flash stimulus used should be recalculated and

given as cd.s.m^-2^.

We apologize for the oversights in the units and calculation. We have made the following corrections accordingly.

[Line 256] A single flashlight (50 cd･s/m^2^) from an LED was used as the light stimulus.

- Line 252 Was fluorescence imaging of retinal tissue sections containing the PFN probe reported? Was there any differences between any of the three probes?

We have previously reported on the imaging of lipid radicals in the retina using NBD-Pen and have added a citation of the relevant paper. NBD-TEEPO was developed for *in vitro* screening, and we believe that NBD-Pen is suitable for *in vivo* imaging.

[22] M. Enoki, S. Shinto, Y. Matsuoka, A. Otsuka, S. Kaidzu, M. Tanito, T. Shibata, K. Uchida, A. Ohira, M. Yamato, K.I. Yamada, Lipid radicals cause light-induced retinal degeneration, Chem. Commun. 53 (2017) 10922–10925. https://doi.org/10.1039/C7CC03387G.

- Line 253 Please verify NBD-Pen concentration used. This is given as 5.0 μmol/kg in Methods (L253) but 50 μmol/kg in Fig 2H Legend (L868).

We apologize for the ambiguity regarding the NBD-Pen concentration in the results and methods. The numbers in the figure legend were incorrect. Therefore, we have corrected them as follows:

[Line 697] (**H**) NBD-Pen (5.0 µmol/kg) was administered intraperitoneally at the end of light exposure.

- Line 287 and Fig S5 Legend. Please define “NSR”.

We have indicated that “NSR” is an abbreviation for “neurosensory retina” in the revised manuscript.

- Line 532 & Line 533. It would probably be better to swap these sentences as Fig 4C shows the arm entry data (L533) before the alternation data (L532).

We agree, and in accordance with this feedback, we have swapped the indicated sentences as follows.

[Line 549] Additionally, spatial working memory was evaluated via the Y-maze test. The number of arm entries did not differ between the two groups (**Fig. 4C**). The percentage of successful arm changes decreased 4 weeks after BCAS occurrence but was preserved by MD administration (**Fig. 4C**).

- Line 595. “MD inhibits retinal damage at ….. a concentration of 1% via ophthalmic administration.” Interestingly, Fig 2D seems to show that retinal damage is also inhibited by 0.45% MD? A comment could be added discussing this.

We agree with this suggestion. Therefore, we have amended this information accordingly.

[Line 601] MD inhibited retinal damage at doses of 100 and 200 μmol/kg (21.1 and 42.2 mg/kg) via oral administration and at a concentration of 0.45 and 1% via ophthalmic administration in a dose-dependent manner.

Comments on Results:

- Fig 2B, D, E. Please define “p.o.”

We have clarified that “p.o.” stands for oral administration in the figure legend.

[Line 698] MD was administered orally (200 µmol/kg, p.o.) 30 min before light exposure (C to H) or using eye drops (1% MD) 30 min before and 5 h after the start of light exposure (C and E).

- Fig 2 data. The authors’ interpretation and reconciliation of the data presented in Fig 2C, D, E could be discussed in greater detail. The resolution of the images provided is not very satisfactory. In particular the retinal histology images (Fig 2C and Fig S3B) are very blurry.

Typically images would look like this:

But the submission images have much poorer resolution:

Was this the limit of the instrumentation?

We apologize for any lack of clarity in the retinol histology imaging. We had uploaded low-resolution images in error. However, in the revised version, we have provided high-resolution data.

- Even with limited imaging quality, Fig 2D appears to show a large (approx. >60%) loss of photoreceptor cell bodies (ONL thickness) following light damage. However, Fig 2E shows only an approximate 25% decline in the photoreceptor-derived ERG a-wave amplitude. This is an important result and needs an explanation. Moreover, no decrease in the ERG b-wave is apparent. As it is believed that the b-wave is generated by bipolar cells subsequent to photoreceptor activation, an explanation should be provided as to why a 60% loss of photoreceptors appears to not have any apparent effect upon inner retinal function. - Representative ERG recording traces should be provided for each treatment condition shown in Fig 2E.

According to Tanito et al. (*Exp Eye Res* 87, 292, 2008), the upper regions of the retina are more affected than the lower regions in the light exposure model, as shown in the figure below. In this study, to evaluate retinal damage, we sectioned the eyeball in the S-I direction and measured the thickness of the ONL between Q and T (**Fig. S4A**), where the damage is most pronounced. Therefore, it is reasonable that the ERG, which evaluates the function of the entire retina, would show less damage because some parts of the retina are less severely affected. We have shown the thickness of the ONL across the entire retina in **Fig. S4B** and provided representative ERG waveforms in **Fig. S4C**.

(Fig. 3B in Tanito *et al. Exp Eye Res* 87, 292, 2008)

**Fig. S4. Evaluation of ONL thickness in a light-induced retinal damage model.**

(**A**) The thickness of the ONL was evaluated at 26 retinal points (A–Z). (**B**) Mean ONL thickness at each defined area. MD was administered orally (p.o.) 30 min before light exposure or using eye drops 30 min before and 5 h after the start of light exposure. The ONL thickness in the most affected regions (Q–T) was quantified in Fig. 2A. *n* = 7 mice, except *n* = 6 mice for eye drop administration. (**C**) Representative electroretinogram waveforms. a- and b-wave amplitudes were quantified in Fig. 2E.

- Fig 2G y-axis “TUNEL^+^ %”. Percentage of what specifically? Total number of cells in the ONL? In the retina? This needs to be clarified.

We apologize for the lack of clarity regarding the percentage shown. We have revised this part accordingly. Fig 2G shows the percentage of TUNEL^+^ cells within the ONL. We have added "/ONL" to the label of the Y-axis.

- Fig 3B “C3^+^ area/RPE”. What antibody was used to stain C3? It is not listed in Section 2.15. How was “/RPE” quantified? Is it RPE area? How was this measured? Surely the quality of the images shown in Fig 3B would not allow quantification of the RPE?

We have added information about the anti-C3 antibody to the Materials and Methods (line 274). The area of the RPE was determined from DAPI staining images. We have replaced the images with contrast-enhanced images to highlight the area of RPE.

- Fig 4E. Please provide a more representative IBA-1 Western Blot image i.e. where the staining intensity corresponds to the small (approx. 1.6 fold) increase (BCAS vehicle vs sham, second panel). The image provided shows an almost infinite increase.

We have replaced the image with a more representative one.

- Fig S6B and Legend. Glutamine synthetase (GS^+^ area) is calculated, but the methodology is not described. The GS antibody is not listed in Section 2.15. A representative image of GS labelling should be amended to Fig S6B.

We have added information about the anti-GS antibody to the Materials and Methods section (line 276) and representative images of GS labeling to Fig. S8B.
